# A new method for deep learning detection of defects in X-ray images of pressure vessel welds

**DOI:** 10.1038/s41598-024-56794-9

**Published:** 2024-03-15

**Authors:** Xue Wang, Feng He, Xu Huang

**Affiliations:** School of Electrical Engineering, Chongqing University of Science and Technology, HuXi Street, Chongqing, 401331 China

**Keywords:** Pressure vessel welds, X-ray images, DC-GAN, Defect segmentation, U-Net, Energy science and technology, Mathematics and computing

## Abstract

Given that defect detection in weld X-ray images is a critical aspect of pressure vessel manufacturing and inspection, accurate differentiation of the type, distribution, number, and area of defects in the images serves as the foundation for judging weld quality, and the segmentation method of defects in digital X-ray images is the core technology for differentiating defects. Based on the publicly available weld seam dataset GDX-ray, this paper proposes a complete technique for fault segmentation in X-ray pictures of pressure vessel welds. The key works are as follows: (1) To address the problem of a lack of defect samples and imbalanced distribution inside GDX-ray, a DA-DCGAN based on a two-channel attention mechanism is devised to increase sample data. (2) A convolutional block attention mechanism is incorporated into the coding layer to boost the accuracy of small-scale defect identification. The proposed MAU-Net defect semantic segmentation network uses multi-scale even convolution to enhance large-scale features. The proposed method can mask electrostatic interference and non-defect-class parts in the actual weld X-ray images, achieve an average segmentation accuracy of 84.75% for the GDX-ray dataset, segment and accurately rate the valid defects with a correct rating rate of 95%, and thus realize practical value in engineering.

## Introduction

X-ray images of pressure vessel welds are critical for finding interior weld faults and are required for pressure vessel manufacture, final inspection, and rework. Because digital X-ray image technology is becoming more common, defect detection technology based on picture segmentation has recently attracted greater attention. The nature, size, and the amount of faults can determine the weld’s present grade, providing a crucial basis for the pressure vessel’s safe operation. The problematic part can be successfully stripped using fault segmentation.

The methods for defect segmentation by X-ray images of weld seams can be divided into methods based on image processing and methods based on deep learning.

In image processing, related research has been conducted early. In 2012, Shao et al.^1^ suggested a tracking technique to track the weld information to complete defect identification after thresholding was used to partition the weld region in X-ray pictures. In 2016, Chu et al.^2^ accurately measured the specific size of the weld seam by extracting contour features from the X-ray image at the weld joint.

In recent years, Defect algorithms based on image processing have recently been the subject of more investigation. In 2021, Amir Movafeghi et al.^3^ used three algorithms based on non-local regularization to improve the detection of weld defects by implementing image enhancement on GDX-ray weld images to improve detection accuracy. In 2022, Doaa Radi et al.^4^ designed an image preprocessing, convolution, horizontal filter, and vertical filter series of image processing methods to segment defects and backgrounds with high method efficiency; etc.

Weld defect segmentation using image processing has the benefit of requiring fewer samples, but in actual applications, CPU time is consumed, end-to-end processes cannot be achieved, segmentation models are less adaptable, detection accuracy is unstable, and detection efficiency is low. The emphasis of recent research, however, has shifted to deep learning-based weld defect segmentation, which has an end-to-end procedure, improved model generality, easier deployment, and high detection efficiency.

In 2020, Ajmi et al.^5^ proposed the classification of weld images in GD-Xay images based on improved AlexNet and migration learning in the study of classification with some effect.

In 2021, Fan et al.^6^ who addressed the problem of insufficient samples of laser welding defects, proposed an ACGAN to generate dummy data like the distribution of real defect features. The model proposed in the paper achieves excellent classification results in defect classification. Yang et al.^7^ used improved U-Net for image segmentation of augmented training samples and finally achieved satisfactory accuracy on the GD-Xay dataset.

In 2022, Yang et al.^8^ designed a feature fusion module embedded in U-Net, and designed the proposed hybrid loss function of binary cross entropy (BCE) and Dice. Ai Jiangsha et al.^9^ proposed an additional supervised method based on CycleGAN-ES to generate synthetic defect images using a small number of extracted defect images and manually drawn labels, where ES is used to ensure the learning of bi-directional mappings corresponding to labels and defects. Hu et al.^10^ proposed an improved pooling method based on grey scale adaptive can increase the extraction range of welding defect features.

In a related study in 2023, Wang et al.^11^ proposed a comprehensive method for X-Ray weld defect detection by nonlinearly enhancing the image with a sinusoidal function, and then using a clustering algorithm in the region of interest (ROI) to segment the defects in the SDR. Xie et al.^12^ carried out a study on defect detection of weld Xray images in the private dataset Camera-Welds, applying Convolutional Neural Networks (CNN) for feature extraction and prototype generation of the embedding module to have low computational cost and high generalization of the images, and using Prototype Networks (PNs) of the prototyping module to reduce the effect of the domain drifts induced by different materials or measurements. Li et al.^13^ proposed a defect lightweight detection model based on hybrid expansion convolution and cross-layer feature fusion of high-resolution X-ray welding images to achieve defect detection in radiographs of solid rocket motors.

Weld defect detection using GDX-ray is a widespread method, however, the weld defects inside this dataset have the following problems: the number of samples is small, the number of publicly available samples of pressure vessel weld defects is scarce in practice, and the current models based on small samples are all suffering from problems such as insufficient training, model underfitting, and difficulty in training; the distribution of defect classes is unbalanced, and the frequency of weld defects of different types is not balanced. This may be due to various factors such as welding operations, material quality, equipment condition, etc. This imbalance may lead to quality, productivity inefficiencies and safety hazards, making defect segmentation based on this dataset very difficult. Although the sample expansion by GAN and the defect segmentation by more advanced techniques like U-Net have yielded some promising results, the following issues still exist: The quality and quantity of sample creation are unpredictable in the sample expansion since there is still a lack of an effective evaluation model for generating samples; the sample expansion model also needs development. There is still a need for image segmentation. To accurately describe the type, quantity, and area of defects and thus accomplish practical work such as weld quality rating, a framework with a better segmentation effect is required. This framework must not only have a good segmentation effect on the GDX-ray dataset but also have good segmentation capability on X-ray images of weld seams in actual engineering.

The general provisions are as follows, according to the Chinese industry standard NB-T 47013.2-2015 for pressure vessel weld fault rating:

1. Only round defects are allowed in Class I welded joints.
2. Class II and Class III welded joints are allowed to have only round defects and strip defects.
3. Round defects and strip defects exist in the round defect assessment area at the same time, a comprehensive rating is required.
4. The quality level of defects in welded joints rated more than level is always set as level.

The literature^14,15^ has more material that is more comprehensive. The key to the X-ray rating of pressure vessel welds, in accordance with the practical requirements of inspection, is to distinguish between round, strip, and other faults and to acquire precise information about their area size and distribution characteristics. As a result, automatic intelligent rating is possible.

In this paper, we propose a research idea: using GAN network to expand the existing samples of weld defects and using semantic segmentation model to accurately segment the weld defects for the purpose of rating. The main contributions and innovations of this paper are as follows:

1. In this paper, a DA-DCGAN pressure vessel weld defect sample generation model is designed, in which a self-attentive generator and a two-channel discriminator are proposed to solve the problems of small size of the existing pressure vessel weld defects, lack of sample quantity, and uneven distribution of samples, resulting in the generation of results that lose the structure and are not real and of poor quality.
2. To solve the problem of complex edges and poor segmentation effect of pressure vessel weld defects. A weld defect segmentation network based on multi-scale even-convolution attention U-Net network is proposed. A 2 × 2 even convolution is used instead of the commonly used 3 × 3 convolution, and a 4 × 4 convolution branch is added to extract feature information within a larger scale; in addition, CBAM is embedded in the coding layer to improve the segmentation accuracy.

## Materials and methods

The techniques for sample enlargement often rely on GAN (Generative Adversarial Networks)^15^, VAE (Variational Autoencoder)^16^, and Autoregressive Models (AM)^17^, among other models. GAN is a generative model trained by a game of generators and discriminators. The disadvantage of VAE training is that it is unstable and difficult to interpret; nonetheless, the effect and variety of the generated samples are worse than those of GAN; similarly, AM production speed is slow, and its diversity is poorer than that of the previous two approaches. If a GAN structure with good improvement is created, it is possible to generate weld images that meet the diversity and complexity of weld faults.

### GAN-based baseline model selection and evaluation of generated images

#### Generate evaluation criteria based on FID, LPPS, and MS-SSIM weighted fusion images

To evaluate the quality, diversity and stability of image generation in the environment of this paper, a weighted fusion judgment index based on FID-LPPSMS-SSIM is designed. The judgment is mainly performed in terms of the diversity and perceptual similarity of the generated images.

The FID (Fréchet Inception Distance)^18,19^ metric calculates the Fréchet distance of the Gaussian distribution of two distributions, which reflects the difference between the two distributions, and this metric is mainly used to generate a diversity discrimination of image quality. This can be expressed by the following calculation: Formula (1)

1 $$ FID(x,g) = \parallel \mu_{x} - \mu_{{\mathbf{g}}} \parallel + Tr\left( {{\Sigma }_{x} + {\Sigma }_{{\mathbf{g}}} - 2\sqrt {{\Sigma }_{x} {\Sigma }_{{\mathbf{g}}} } } \right) $$

In Formula (1): $x$ denotes the real original image and $g$ denotes the generated image, $\mu_{x}$ and ${\Sigma }_{x}$ denote the mean and covariance matrices of the high-dimensional features extracted from the real image, respectively.$\mu_{g}$ and ${\Sigma }_{g}$ denote the mean and covariance matrices of the high-dimensional features extracted from the generated images, respectively. $Tr$ denotes the trace of the matrix. FID can reflect the diversity of the generated images.

2 $$ d(x_{1} ,x_{2} ) = \sum\limits_{l} {\frac{1}{{H_{l} W_{l} }}} \sum\limits_{h,w} {\parallel w_{l} \odot (\hat{y}_{1hw}^{l} - \hat{y}_{2hw}^{l} )\parallel_{2}^{2} } $$

To reflect the image perceptibility, the image perceptual similarity is utilized. LPSIPS^20,21^ (Learned Perceptual Image Patch Similarity) can be expressed by the following calculation, Formula (2):

The $w_{l} \in {\mathbb{R}}^{{c^{l} }}$ is a scaled vector. LPIPS has a high accuracy and reliability in detecting subtle differences in the images generated by GAN.

MS-SSIM^22^ (Multi-Scale Structural Similarity Index) is a metric for evaluating image quality, which is an extension of the Structural Similarity Index (SSIM) on multiple scales.MS-SSIM introduces multiple scales on top of SSIM to better capture the detailed information in images. It can be expressed by the following calculation, Formula (3):

3 $$ SSIM\left( {x,y} \right) = \left[ {l_{M} \left( {x,y} \right)} \right]^{{\alpha_{M} }} \cdot \prod\limits_{j = 1}^{M} {\left[ {c_{j} \left( {x,y} \right)} \right]^{{\beta_{j} }} } \cdot \left[ {s_{j} \left( {x,y} \right)} \right]^{{\gamma_{j} }} $$

The $\alpha$*,* $\beta$*,* $\gamma$ is used to adjust the weights of each component.

The designed weighted fusion determination index is $F_{total}$, taking into account the influence factor of FID − *F*_*f*_, the influence factor of LPSIPS − *F*_*l*_ and the influence factor of MS-SSIM − *F*_*M*_. To maintain consistency with the other two factors, the data calculated by Eq. (1) are normalized to (0–1) and subtracted by 1, all three indicators show that the closer to 1 (0–1), the worse the generation effect. The designed fusion determination indicators can be expressed by the following calculation, Formula (4):

4 $$ F_{total} = \alpha F_{f} + (1 - \alpha )\left[ {\beta F_{l} + (1 - \beta )F_{M} } \right] $$

There are three groups of defects, including bar-shaped defects, circular defects, and other forms of defects, that make up the network input. The score acquired from the formula to be trained (3) is the calculated value, the loss function is the difference value between the two, and the average score of the two experts is used as the standard value for each created image (Table 1). The following calculation can be used to express the ultimate best value expression in Formula (5):

5 $$ F = 0.43F_{f} + 0.57\left( {0.55F_{l} + 0.45F_{M} } \right) $$

**Table 1:** Independent expert scoring table.

Score	Defect form	Defect size	Background	Intuitive feeling
95–100	Match	Normal	Match	Real
90–94	Match	More normal	Match	More real
85–89	Better match	More normal	Match	Basically true
80–84	Better match	More normal	Better match	Basically true
75–79	Basically match	Basically normal	Basically match	Not very true
70–74	Basically match	Problems	Problems	Not very true
60–69	Problems	Problems	Problems	Not very true
60	False	False	False	Unreal

### Quantitative comparison and analysis of different generative models

In this section, GAN, DCGAN, WGAN and LSGAN are trained and tested using the same dataset and experimental environment. The specific results are shown in Table 2.

**Table 2:** Comparative results of generated models.

Models	FID	LPIPS	MS-SSIM
GAN	296.4	0.475	0.704
DCGAN	283.7	0.428	0.689
WGAN-GP	284.2	0.419	0.674
LSGAN	285.5	0.421	0.678

From the experimental results, DCGAN, WGAN-GP and LASGAN, as improved models of GAN, have significant improvement in FID, LPIPS and MS-SSIM metrics compared to the original GAN.

By changing the network structure, DCGAN has no pooling layer and up-sampling layer in the whole network, and adopts step convolution instead of up-sampling to increase the stability of training, DCGAN has a very good performance in the three evaluation indexes, especially the FID value of the image evaluation indexes has been greatly improved, which indicates that the quality effect of the image generated by DCGAN is better than that of the other generation models. However, in pressure vessel weld defect expansion applications, the performance of DCGAN still needs to be improved, and the structural information and texture content information of the generated images need to be further enhanced. Therefore, the ability of DCGAN to extract structural and content features needs to be enhanced to improve the generated results.

### DA-DCGAN-based defect generation model

Unrealistic defect morphology, distribution, and background are still concerns in the defect images produced by the base DCGAN model^23^. To address these issues, we provide a Double Attention based DCGAN (DA-DCGAN) model in this research. This model includes an attention mechanism and builds a Self-Attention Generator (SAG) and a Double Attention Discriminator (DAD).

#### The architecture of DA-DCGAN network

**Figure 1:**
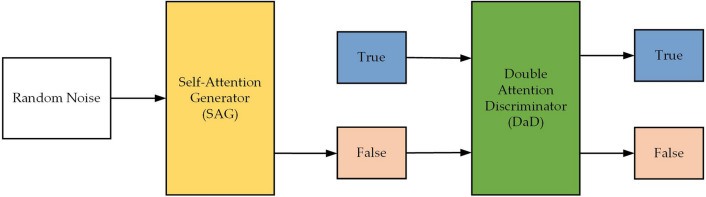
DA-DCGAN network structure.

The designed of DA-DCGAN network structure is displayed in Fig. 1. The DA-DCGAN developed in this study has a two-channel discriminator, DaD, and a generator, SAG, based on the self-attention mechanism. The attention module is used in DaD to learn significant regions and features in images to better distinguish between real and generated images and to more thoroughly evaluate the quality of the generated images. The design idea for SAG is to consider generating more realistic images, reducing overfitting, and appropriately increasing the training speed.

#### Generator of self-attentive mechanisms

**Figure 2:**
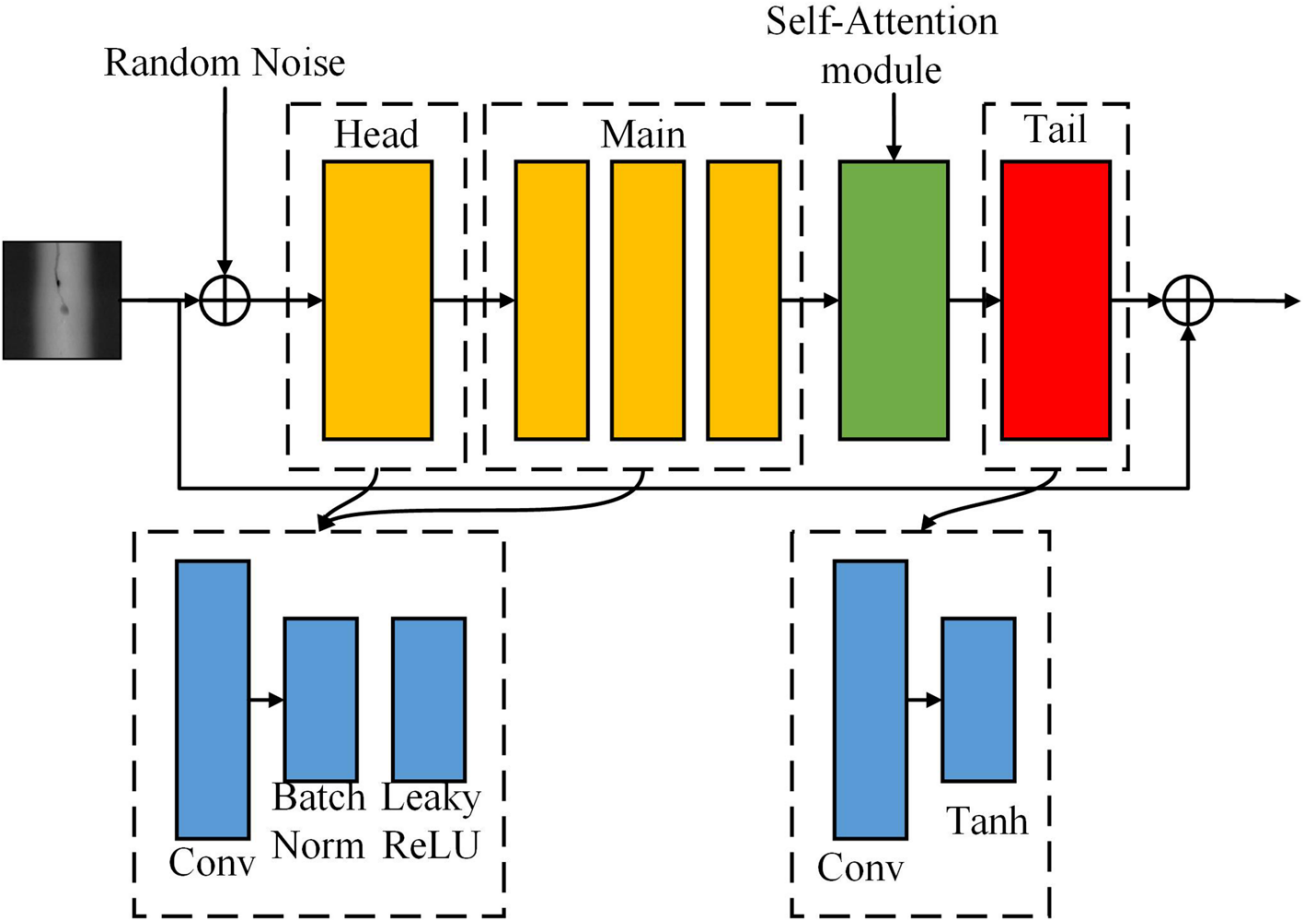
Structure of SAG.

The structure of the self-attention generator is displayed in Fig. 2:

The convolutional network generator uses a whole structure, including a head, main body, self-attention module, and tail. To convert the number of input picture channels to C, the head portion of the generator developed in this study consists of a convolutional layer, a batch norm layer, and many LeakyReLU activation functions. The body portion has numerous convolutional layers, a BatchNorm layer, and LeakyReLU activation functions, and it is identical to the head in appearance. The convolutional layer and Tanh activation function make up the tail portion, which maps the output values to the (− 1, 1) range. Utilizing a fully convolutional network structure, the generator can produce images of any shape or size. The flexibility and usefulness of the generator are significantly increased by the structure’s ability to produce images of varied sizes and scales adaptively in response to the input noise size.

#### Dual-channel based discriminator

**Figure 3:**
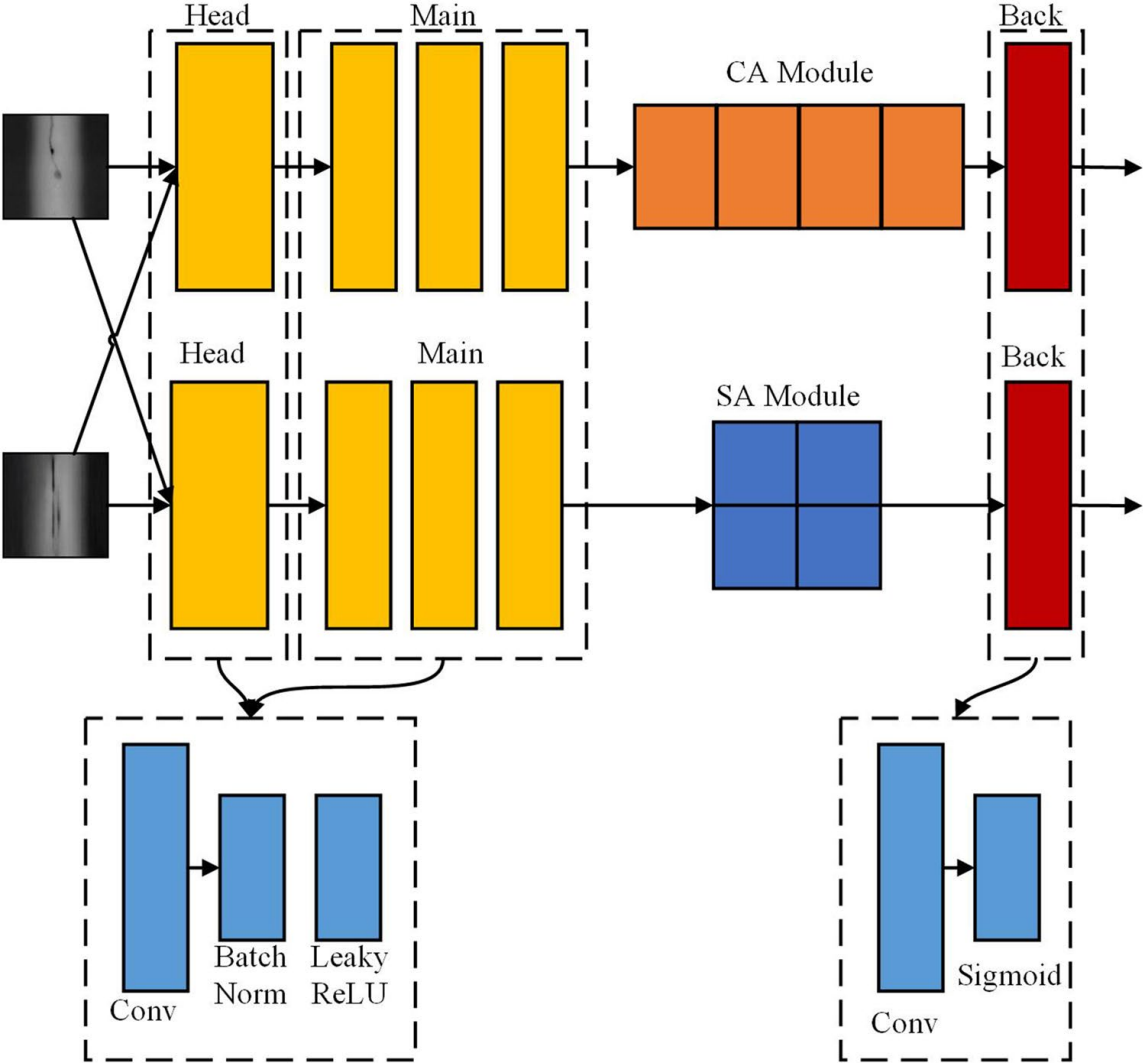
Structure of the two-channel attention discriminator.

Based on the two-channel attention discriminator structure^24^, as shown in Fig. 3, the dual-channel attention discriminator structure mainly consists of two parts, which are Spatial Attention Layout Discriminator (SALD) and Channel Attention Content Discriminator (CACD). To increase the generator’s capacity to produce high quality photos, these two sections concentrate on the content fidelity and texture layout distribution fidelity of the created images, respectively. The generator has a shallow network topology with a head and main body that are used by the two-channel discriminator. The body is built using several parameter-specific convolutional blocks and the LeakyReLU activation function, whereas the head is made up of a single layer of parameter-specific convolutional layers.

The channel attention module in CBAM is used as the feature extraction component of the content discriminator CACD^25^, and the spatial attention module in CBAM is used as the feature extraction part of the layout discriminator SALD. The features that pass the adjustment are obtained using the hybrid attention method by multiplying the channel attention map by the initial input features. A change like this can enhance the image’s ability to extract important textural characteristics and semantic information, assisting the discriminator in more precisely determining how realistic the created image is. A chunking discriminator is used to score the layout of the input image for the layout discriminator SALD. To determine a layout score for the entire image, the image is divided into several blocks, with each block subject to a layout judgment. Finally, there are only 1 feature channels output using a convolutional layer, and the value at each point represents the likelihood that the layout decision will be accurate for that region.

#### Generating defective image analysis based on DA-DCGAN

**Figure 4:**
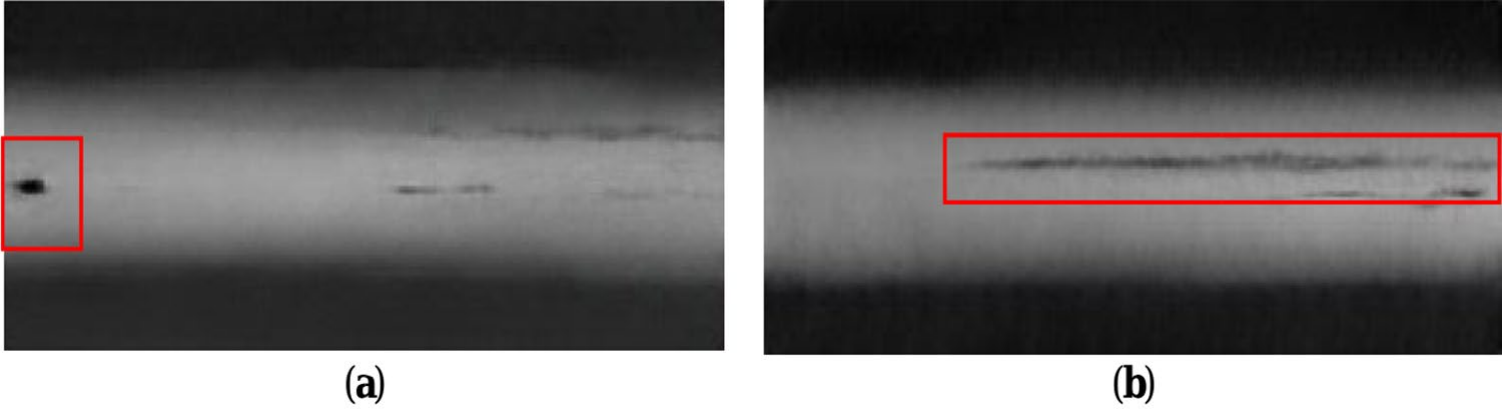
Generated typical defect map (**a**) Generated round defects; (**b**) Generated bar defects.

Typical defects generated based on DA-DCGAN are displayed in Fig. 4: red boxes are typical defects generated. Evaluation metrics for the generated images are shown in Table 3.

**Table 3:** Comparison results of generated models.

Models	FID	LPIPS	MS-SSIM	F
DCGAN	283.7	0.428	0.689	0.632
+ SAG	279.1	0.406	0.677	0.624
+ SALD	280.2	0.397	0.674	0.620
+ CACD	279.3	0.413	0.668	0.625
Ours	277.6	0.391	0.658	0.614

The quantitative evaluation of the DA-DCGAN model proposed in this paper was carried out using the FID metric as the evaluation index. As shown in Table 2, compared with the baseline model DCGAN, the FID values of the SAG model with the introduction of self-attention mechanism, the SALD model with spatial attention mechanism and the CACD model with channel attention have decreased, which shows that all the three modules are effective in improving the sample generation capability in terms of the quality assessment metrics of image generation. In addition, the diversity of generated samples was also evaluated, and it was found that the three models improved a certain image diversity accordingly, indicating that the proposed modules can improve the diversity of generated images to a certain extent.

The defects created using the method described in this paper have a high degree of confidence in their defect morphology and genuine defect kinds, with no grid interference in the background and a suitable distribution, according to the aforementioned analysis.

### MAU-Net based defective semantic segmentation algorithm

#### Multi-scale convolutional coupling architecture

Currently, the main body of the U-Net network still uses 3 × 3 convolutional kernels for feature extraction. To reduce the computational effort without losing accuracy, this paper adopts a smaller size of even convolutional kernel to extract image information instead of the original 3 × 3 convolutional.

**Figure 5:**
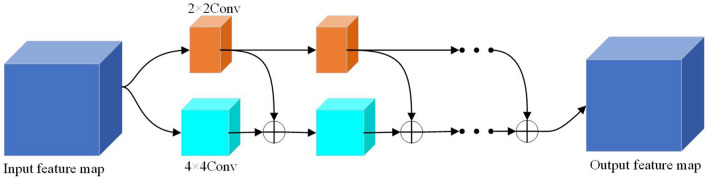
Multiscale even convolution module.

Multiscale Even Convolution Module is displayed in Fig. 5.

A 2 × 2 even convolution kernel is used to extract image information^26,27^. Meanwhile, to solve the pixel shift problem brought about by the even number of convolutional kernels, a symmetric filling method is used to expand the perceptual field. In addition, to avoid excessive parameters and complexity introduced by multi-scale feature fusion, this study adds a new layer of 4 × 4 even convolutional coding network outside the main part of the segmentation network for extracting image information separately. In each layer, a larger-scale 4 × 4 even convolutional kernel is used to extract the image information, and the feature maps to be segmented are symmetrically filled to avoid the pixel shift problems. Finally, the acquired information is passed to the subject network for the next pooling step by stitching. This approach can acquire image information more comprehensively while avoiding the parameter explosion problem introduced by multi-scale feature fusion.

Finally, the acquired information is passed to the subject network for the next pooling step by stitching. This approach can acquire image information more comprehensively while avoiding the parameter explosion problem introduced by multi-scale feature fusion.

#### The design of MAU-Net network

**Figure 6:**
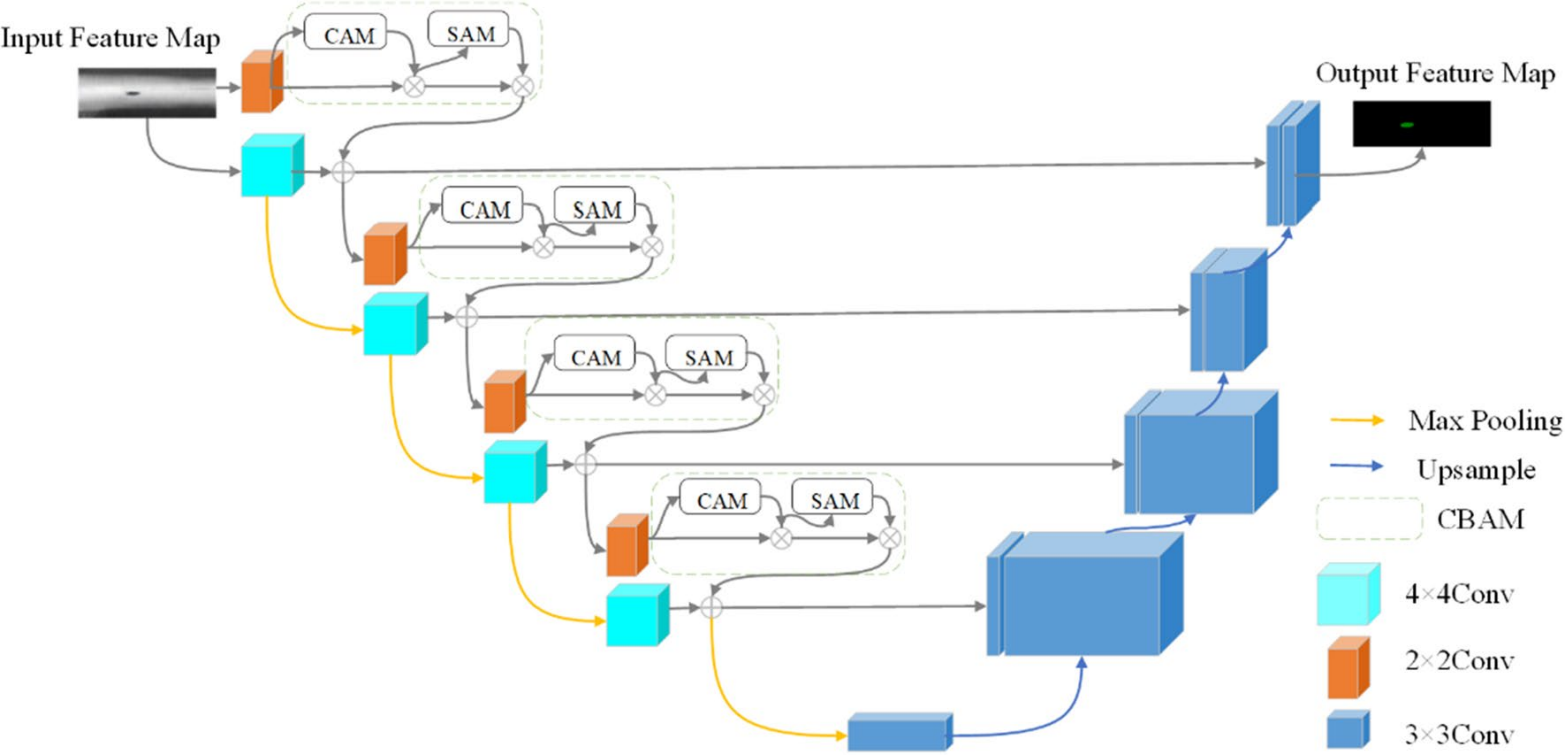
The structure of MAU-Net.

The designed of MAU-Net network structure is displayed in Fig. 6.

The MAU-Net network employs several optimization methods to improve the extraction of weld defect feature information. First, an even convolutional kernel of size 2 × 2 is used in the main part of the network, and a 4 × 4 even convolutional path parallel to the 2 × 2 even convolution is also created at the coding end to fuse feature information within different perceptual fields, while eliminating the pixel shift problem caused by the even convolutional kernel. A residual module is incorporated to create a deep residual network to further improve the extraction and representation of weld defect feature information. A CBAM attention module is embedded after the 2 × 2 even convolution module to effectively segment the target regions in the weld samples. The overall de-sign effect is to achieve more accurate segmentation without significantly increasing the additional arithmetic overhead.

### Ethical approval

This study did not involve any human participants and thus did not require ethical approval.

## Experiments

### Experimental software and hardware configuration

The deep learning framework PyTorch was used to implement the model and the experiments. The hardware and software configurations are shown in Table 4.

**Table 4:** The hardware and software configurations.

Parameters	Specifications
CPU	Intel Core i5-9600KF@ 3.70 GHz
GPU	Nvidia GeForce RTX 3060
RAM	16 GB × 2
OS	Windows 10
Deep learning tutorial	Keras

### Datasets

One of the most popular publicly accessible datasets of X-ray images, the GDX-ray dataset^28^, provides more conclusive results for confirming the detection effect. The dataset contains 19,407 X-ray pictures altogether. Five categories of X-ray images are present in the database: castings, welds, luggage, natural objects, and setups. The sample of weld flaws includes 88 of them, which are grouped into three series. The W0001 series, which has 20 complete weld samples, is used in this paper as the dataset for training the weld defect sample expansion model.

### Experiments

The data used for the experiments in this paper is an expanded pressure vessel weld defect X-ray data set of 1500 weld defect samples, including 500 each for circular defects, strip defects, and other defects. In the algorithm model used in this experiment, the number of batches is set to 4, the maximum learning rate is set to 1e−2, the number of training iterations is 300, the number of channels is 4, the decay rate of weights is 1e−4, and the model is trained using the SGD optimizer.

#### Ablation study

To compare the segmentation effect of MAU-Net on defects in this paper, mIOU, mPa, mPrecision, and mRecall are used as evaluation metrics for the experiments. Where mIOU denotes the average of the intersection and concurrency ratios for each class, mPa denotes the category average pixel accuracy, mPrecision denotes the value obtained by averaging the precision rates for each class, and mRecall is the value obtained by averaging the recall rates for each class. The formulas are shown below:

6 $$ mPrecision = \frac{1}{k + 1}\sum\limits_{k + 1}^{k} {\frac{TP}{{TP + FP}}} $$

7 $$ mRecall = \frac{1}{k + 1}\sum\limits_{k + 1}^{k} {\frac{TP}{{TP + FN}}} $$

8 $$ mIOU = \frac{1}{k + 1}\sum\limits_{k + 1}^{{\text{k}}} {\frac{TP}{{TP + FN + FP}}} $$

9 $$ mPa = \frac{1}{k + 1}\sum\limits_{k + 1}^{1} {\frac{TP + TN}{{TP + TN + FP + FN}}} $$

A total of six models are compared in Table 4, namely the standard U-Net, the standard Deeplabv3, the standard PspNet, the standard U-Net with CBMA added to the coded convolution only, the standard U-Net with 3 × 3 convolution replaced by 2 × 2 even convolution, and the method in this paper. The results are shown in Table 5.

**Table 5:** Comparative results of ablation study (%).

Methods	mIou	mPa	mPrecision	mRecall
1. U-Net	66.72	72.34	75.97	74.34
2. Deeplabv3	73.54	76.23	79.52	78.36
3. PspNet	71.23	75.38	78.75	77.11
4. U-Net + CBMA	71.12	75.32	78.43	77.33
5. U-Net + Conv2 × 2	68.26	73.11	76.24	76.29
6. MAU-Net	77.36	79.63	84.75	83.45

As shown in Table 4, the mIou, mPA and mPrecision of segmented images in model 5 in-creased to 68.26%, 73.11% and 76.24%, respectively; the mIou, mPA, and mPrecision of seg-mented images in model 4 showed a more significant improvement compared to model 4. Models 4 and 5 showed significant improvement over the standard U-Net model. mAU-Net had much better metrics for each parameter than all the other models. This proves the effectiveness of the algorithm.

#### Comparative experiment

**Figure 7:**
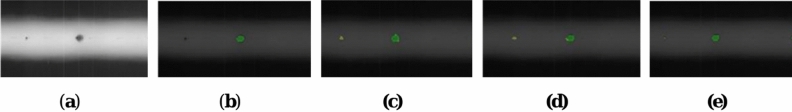
There are comparisons between different models for the segmentation of circular defects inside the dataset. (**a**) Original image of circular defect; (**b**) U-Net, (**c**) PspNet, (**d**) DeeplabV3, (**e**) MAU-Net.

**Figure 8:**
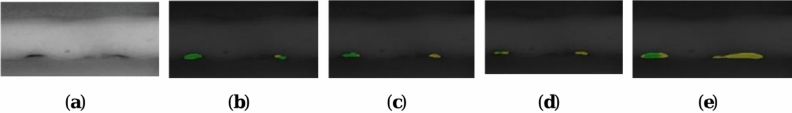
There are comparisons between different models for the segmentation of other defects inside the dataset. (**a**) Original image of other defect; (**b**) U-Net, (**c**) PspNet, (**d**) DeeplabV3, (**e**) MAU-Net.

**Figure 9:**
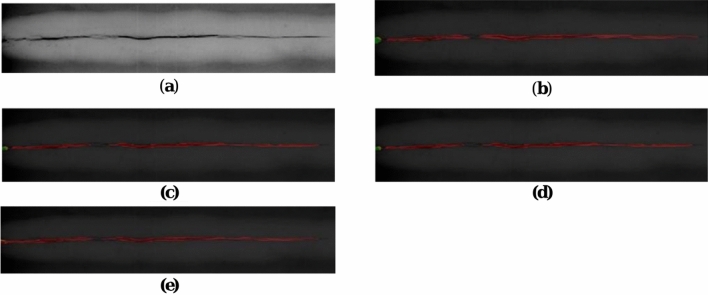
There are comparisons between different models for the segmentation of bar defects inside the dataset. (**a**) Original image of bar defect; (**b**) U-Net result, (**c**) PspNet result, (**d**) DeeplabV3 result, (**e**) MAU-Net.

The segmentation of circular and bar-shaped defect images within the dataset is performed using the MAU-Net in comparison to several models of U-Net, Deeplabv3, and PspNet. The green color on the segmentation graph denotes circular flaws. Other faults are shown in yellow. Bar-shaped flaws are indicated in red. Among them, Fig. 7 shows the circular defect segmentation maps; Fig. 8 shows other defect segmentation maps; and Fig. 9 shows the bar defect segmentation maps.

The comparison experiments were carried out for a total of fault types—round, bar, and other types—identified by Chinese industry standards for rating. The original figure has a total of three flaws of various proportions. Only this paper’s method provides correct identification for the common medium-sized defect on the right; for the bar-shaped defect, there is little difference between all methods; for other defects, some methods have incorrect type determination, and some methods produce large size errors, but this paper’s method maintains accuracy for the very small size defect on the far left.

Tables 6 and 7 present a comparison of IOU and Pa for the defects in the dataset.

**Table 6:** IOU results of different models.

Methods	Others	Round defects	Bar defects	Average
U-Net	0.34	0.52	0.79	0.55
Deeplabv3	0.40	0.56	0.96	0.64
PspNet	0.40	0.53	0.90	0.61
MAU-Net	0.57	0.66	0.85	0.69

**Table 7:** Pa results of different models.

Methods	Others	Round defects	Bar defects	Average
U-Net	0.50	0.50	0.90	0.63
Deeplabv3	0.52	0.61	0.92	0.68
PspNet	0.53	0.52	0.92	0.66
MAU-Net	0.60	0.65	0.91	0.72

#### Testing of X-ray defect pictures under real working conditions

The X-ray images are properly cropped to eliminate numerous sample identifying characters before being added to the model described in this paper for semantic segmentation, which extracts bars, circles, and other flaws. The photos were obtained in the field under real-world working conditions. As can be seen from the actual images, the contrast of the X-ray images of the actual welds is not as strong as it was in the training dataset. Additionally, the photos are subject to contrast variations, smear pseudo-defects, electrostatic interference images, and other factors because of field operations, instrument quality, etc.

**Figure 10:**
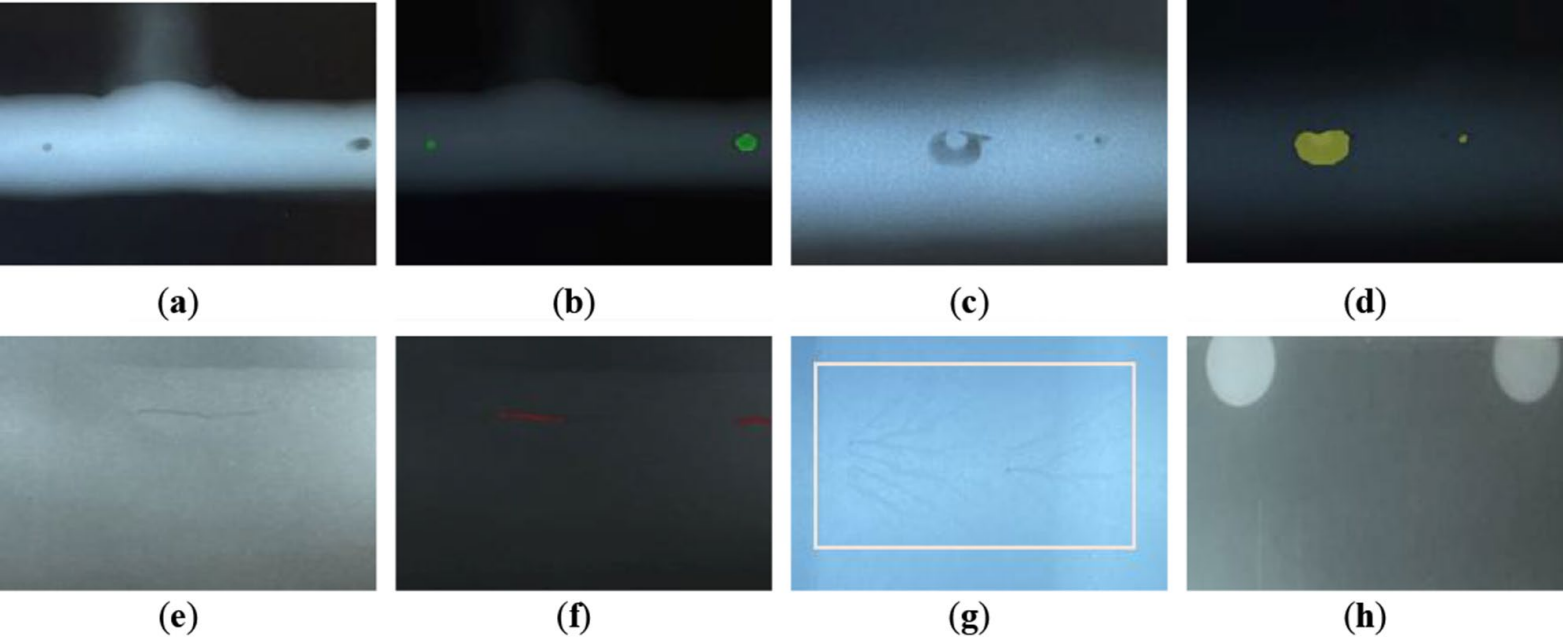
There are results of on-site X-ray image inspection. (**a**, **b**): The original picture of the circular defect found in the T-joint of the pressure vessel and the segmentation effect; (**c**, **d**): Other defects (pinch bead defects) of the original and segmentation effect; (**e**, **f**): The original picture of slight bar and segmentation effect; (**g**, **h**): Electrostatic interference and taint interference, correctly treated as background in identification, are not counted in the defect rating.

The detection in the field picture is shown in Figure 10.

After semantic segmentation, information on the type and number of defects and the number of pixels contained in the defect can be obtained. After semantic segmentation, information on the type and number of defects and the number of pixels contained in each defect can be obtained. The actual faults may be precisely determined based on the calibration of the actual size and imaging size, and the automatic grading of defects is carried out in accordance with Table 1. In the actual rating work, a total of 100 rating tests were carried out, and Table 8 displays the rating comparison. The pressure vessel weld rating results achieved by the method suggested in this research were quite similar to the expert rating results.

**Table 8:** Sample table of partial weld rating.

Number	Results of the rating of the system	Results of expert ratings
1	Grade IV type: round	Grade type: round
2	Grade no defects	Grade type: pseudo defects
3	Grade type: round, bar	Grade type: round
4	Grade type: round	Grade type: round
5	Grade no defects	Grade type: cracks
6	Grade type: round	Grade type: round
7	Grade no defects	Grade no defects
8	Grade IV type: other	Grade IV type: cracked
9	Grade Type: other	Grade type: unfused
10	Grade No defects	Grade type: cracked
11	Grade IV type: other	Grade IV type: cracked

In this paper, 100 actual weld samples were rated and the rating results were compared with expert ratings. In this rating, 73% of the ratings were correct, 82% of the defects were correctly identified, and the time to evaluate the samples was 15 s. Of the 11 ratings listed, 8 had the same results as the experts’ ratings, and 1 had similar results to the experts’ ratings. In addition, in 9 of these samples, the defect type was the same as the expert’s identification, including the successful rating of the ionization pseudo defect sample. The bead-clamping defect on sample No. 1 was successfully segmented as a circular defect, which is highly susceptible to being determined as a bar defect in manual grading.

## Conclusions

The primary technology for intelligently rating pressure vessel weld faults is examined in this paper. The results of this investigation are illustrated as follows:

The proposed DA-DCGAN uses an attention mechanism primarily on the generator and discriminator, and the generated weld defect images can be judged to conform to the morphology of the weld itself by the evaluation model. This is in response to the current situation of a small number of publicly available datasets and a scarcity of on-site weld defect images.

Based on even convolution, a U-Net semantic segmentation network is created. The enhanced approach widens the perceptual field, stays away from multi-scale feature fusion, which adds excessive complexity and parameters, and solves the pixel shift issue.

Circular and strip-shaped defects can be accurately recognized in the actual defect detection of T-weld diagrams and ring weld diagrams of pressure vessels, and image components that are not defects themselves, like ionization lines and stains, can be shielded. By simultaneously considering the defect type, number of faults, and defect area in the real rating, it has been done to a practical extent.

### Supplementary Information

Supplementary Information.

## Data Availability

All research procedures and data collection activities described in this paper comply with applicable ethical principles and regulations. Individuals participating in this study were fully informed of the research's purpose, processes, and potential risks, and they voluntarily consented to participate in this research project on an informed basis. We will share the raw data, either by making it available in a supplemental document or by storing it in a public repository. This study utilized X-ray images from the GDXray database for nondestructive testing. The database was created by the team led by Professor Gonzalo R. Arce and is described in the paper "GDXray: The database of X-ray images for nondestructive testing"^1^. The dataset can be accessed and downloaded from the following link: http://dmery.ing.puc.cl/index.php/material/gdxray.

## Code availability

The code used in this study is considered for storage in a relevant online repository and will be provided as supplementary material to this paper. For access to the code or further details on implementation, please contact the corresponding author. We are committed to promoting transparency and reproducibility in research, and welcome any inquiries regarding our code repository.
