# Supplementary figures and images for: A new method for deep learning detection of defects in X-ray images of pressure vessel welds (part 2 of 2)
Source: Sci Rep. 2024 Mar 15;14:6312. doi: 10.1038/s41598-024-56794-9 (PMC10943115; doi:10.1038/s41598-024-56794-9)

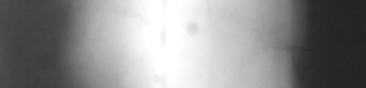

Supplement: Supplementary file 1 — Supplementary Information. [file 41598_2024_56794_MOESM1_ESM.zip › Supplementary/datasets/19.png]

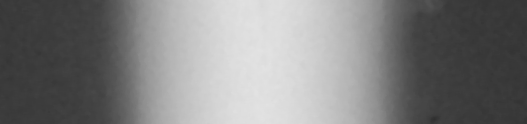

Supplement: Supplementary file 1 — Supplementary Information. [file 41598_2024_56794_MOESM1_ESM.zip › Supplementary/datasets/190.png]

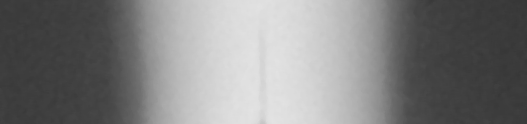

Supplement: Supplementary file 1 — Supplementary Information. [file 41598_2024_56794_MOESM1_ESM.zip › Supplementary/datasets/191.png]

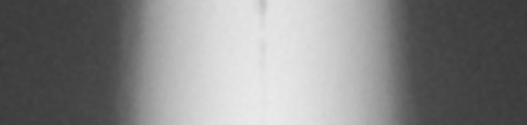

Supplement: Supplementary file 1 — Supplementary Information. [file 41598_2024_56794_MOESM1_ESM.zip › Supplementary/datasets/192.png]

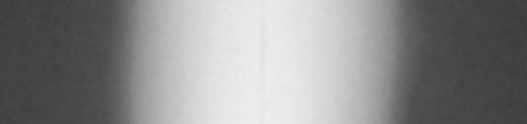

Supplement: Supplementary file 1 — Supplementary Information. [file 41598_2024_56794_MOESM1_ESM.zip › Supplementary/datasets/193.png]

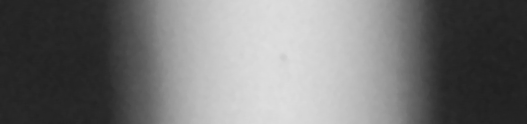

Supplement: Supplementary file 1 — Supplementary Information. [file 41598_2024_56794_MOESM1_ESM.zip › Supplementary/datasets/194.png]

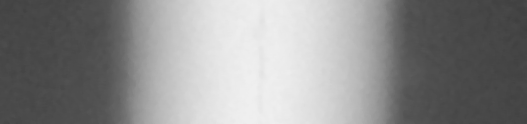

Supplement: Supplementary file 1 — Supplementary Information. [file 41598_2024_56794_MOESM1_ESM.zip › Supplementary/datasets/195.png]

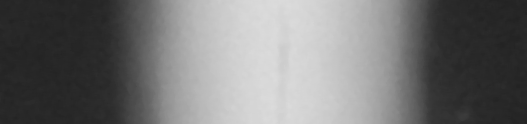

Supplement: Supplementary file 1 — Supplementary Information. [file 41598_2024_56794_MOESM1_ESM.zip › Supplementary/datasets/196.png]

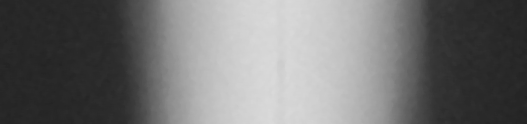

Supplement: Supplementary file 1 — Supplementary Information. [file 41598_2024_56794_MOESM1_ESM.zip › Supplementary/datasets/197.png]

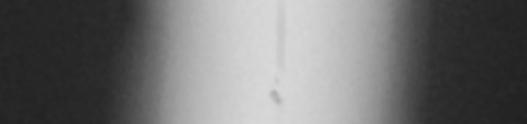

Supplement: Supplementary file 1 — Supplementary Information. [file 41598_2024_56794_MOESM1_ESM.zip › Supplementary/datasets/198.png]

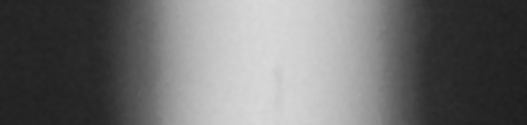

Supplement: Supplementary file 1 — Supplementary Information. [file 41598_2024_56794_MOESM1_ESM.zip › Supplementary/datasets/199.png]

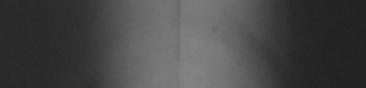

Supplement: Supplementary file 1 — Supplementary Information. [file 41598_2024_56794_MOESM1_ESM.zip › Supplementary/datasets/2.png]

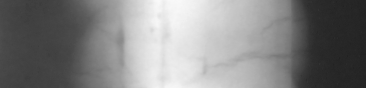

Supplement: Supplementary file 1 — Supplementary Information. [file 41598_2024_56794_MOESM1_ESM.zip › Supplementary/datasets/20.png]

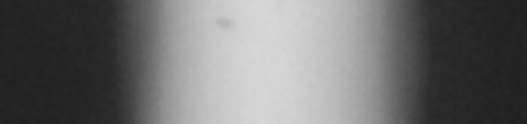

Supplement: Supplementary file 1 — Supplementary Information. [file 41598_2024_56794_MOESM1_ESM.zip › Supplementary/datasets/200.png]

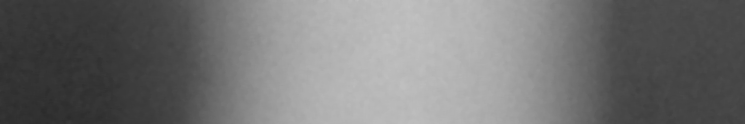

Supplement: Supplementary file 1 — Supplementary Information. [file 41598_2024_56794_MOESM1_ESM.zip › Supplementary/datasets/201.png]

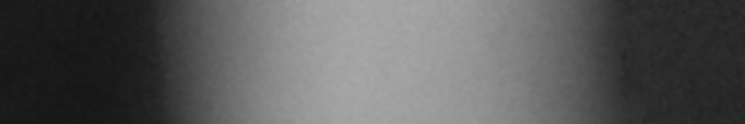

Supplement: Supplementary file 1 — Supplementary Information. [file 41598_2024_56794_MOESM1_ESM.zip › Supplementary/datasets/202.png]

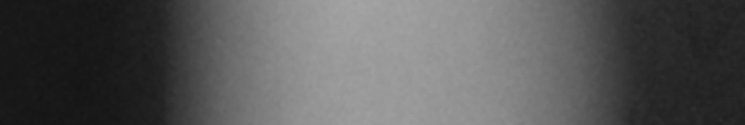

Supplement: Supplementary file 1 — Supplementary Information. [file 41598_2024_56794_MOESM1_ESM.zip › Supplementary/datasets/203.png]

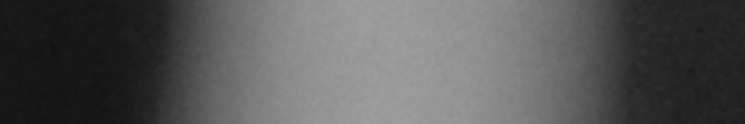

Supplement: Supplementary file 1 — Supplementary Information. [file 41598_2024_56794_MOESM1_ESM.zip › Supplementary/datasets/204.png]

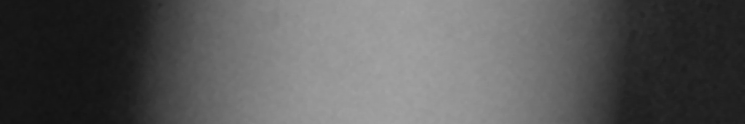

Supplement: Supplementary file 1 — Supplementary Information. [file 41598_2024_56794_MOESM1_ESM.zip › Supplementary/datasets/205.png]

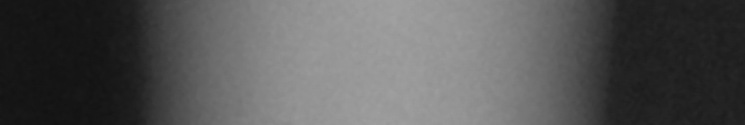

Supplement: Supplementary file 1 — Supplementary Information. [file 41598_2024_56794_MOESM1_ESM.zip › Supplementary/datasets/206.png]

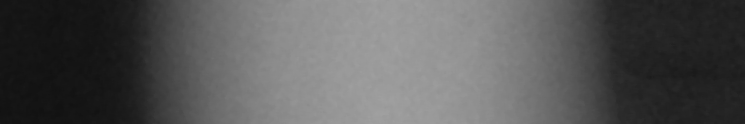

Supplement: Supplementary file 1 — Supplementary Information. [file 41598_2024_56794_MOESM1_ESM.zip › Supplementary/datasets/207.png]

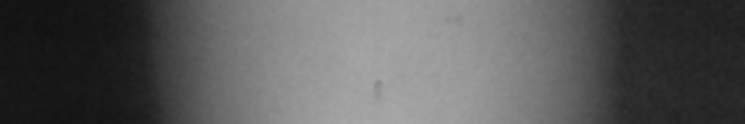

Supplement: Supplementary file 1 — Supplementary Information. [file 41598_2024_56794_MOESM1_ESM.zip › Supplementary/datasets/208.png]

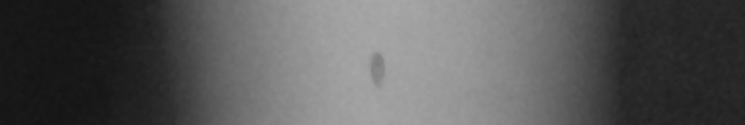

Supplement: Supplementary file 1 — Supplementary Information. [file 41598_2024_56794_MOESM1_ESM.zip › Supplementary/datasets/209.png]

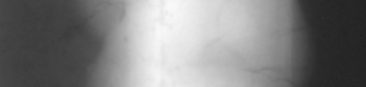

Supplement: Supplementary file 1 — Supplementary Information. [file 41598_2024_56794_MOESM1_ESM.zip › Supplementary/datasets/21.png]

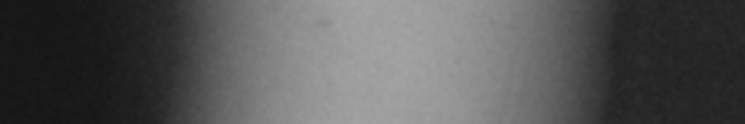

Supplement: Supplementary file 1 — Supplementary Information. [file 41598_2024_56794_MOESM1_ESM.zip › Supplementary/datasets/210.png]

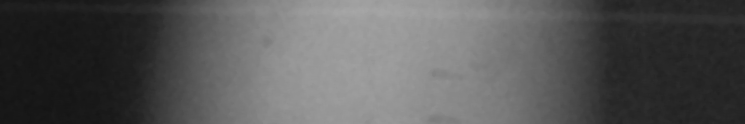

Supplement: Supplementary file 1 — Supplementary Information. [file 41598_2024_56794_MOESM1_ESM.zip › Supplementary/datasets/211.png]

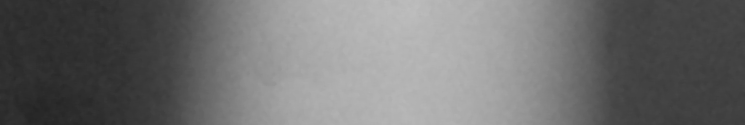

Supplement: Supplementary file 1 — Supplementary Information. [file 41598_2024_56794_MOESM1_ESM.zip › Supplementary/datasets/212.png]

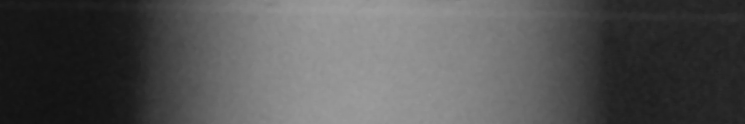

Supplement: Supplementary file 1 — Supplementary Information. [file 41598_2024_56794_MOESM1_ESM.zip › Supplementary/datasets/213.png]

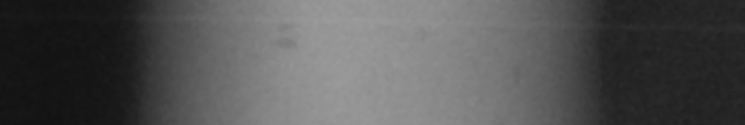

Supplement: Supplementary file 1 — Supplementary Information. [file 41598_2024_56794_MOESM1_ESM.zip › Supplementary/datasets/214.png]

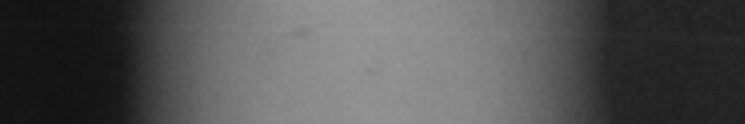

Supplement: Supplementary file 1 — Supplementary Information. [file 41598_2024_56794_MOESM1_ESM.zip › Supplementary/datasets/215.png]

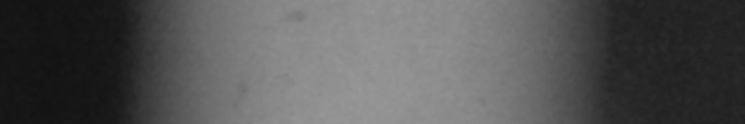

Supplement: Supplementary file 1 — Supplementary Information. [file 41598_2024_56794_MOESM1_ESM.zip › Supplementary/datasets/216.png]

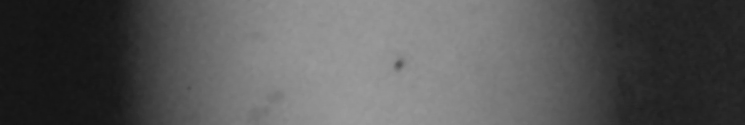

Supplement: Supplementary file 1 — Supplementary Information. [file 41598_2024_56794_MOESM1_ESM.zip › Supplementary/datasets/217.png]

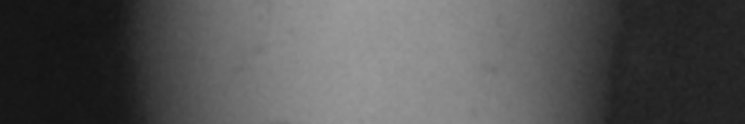

Supplement: Supplementary file 1 — Supplementary Information. [file 41598_2024_56794_MOESM1_ESM.zip › Supplementary/datasets/218.png]

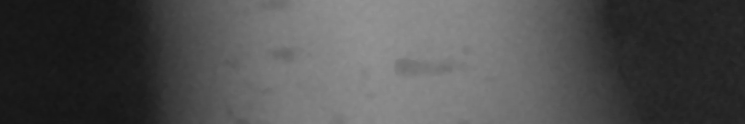

Supplement: Supplementary file 1 — Supplementary Information. [file 41598_2024_56794_MOESM1_ESM.zip › Supplementary/datasets/219.png]

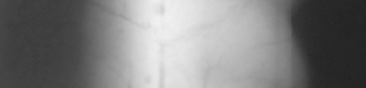

Supplement: Supplementary file 1 — Supplementary Information. [file 41598_2024_56794_MOESM1_ESM.zip › Supplementary/datasets/22.png]

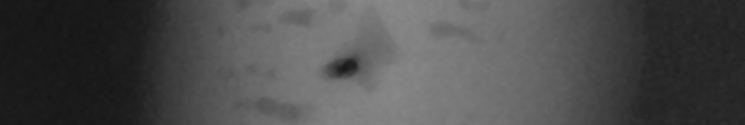

Supplement: Supplementary file 1 — Supplementary Information. [file 41598_2024_56794_MOESM1_ESM.zip › Supplementary/datasets/220.png]

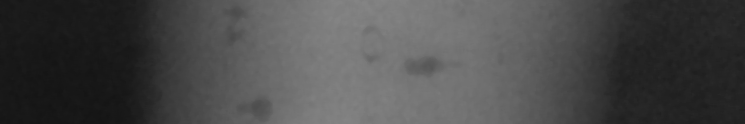

Supplement: Supplementary file 1 — Supplementary Information. [file 41598_2024_56794_MOESM1_ESM.zip › Supplementary/datasets/221.png]

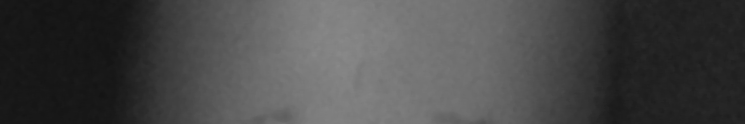

Supplement: Supplementary file 1 — Supplementary Information. [file 41598_2024_56794_MOESM1_ESM.zip › Supplementary/datasets/222.png]

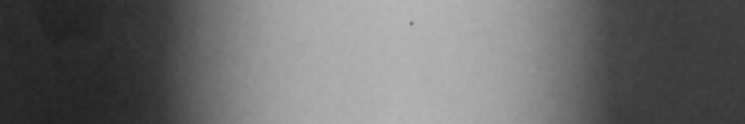

Supplement: Supplementary file 1 — Supplementary Information. [file 41598_2024_56794_MOESM1_ESM.zip › Supplementary/datasets/223.png]

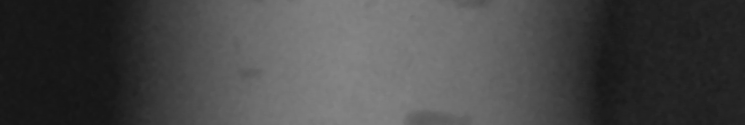

Supplement: Supplementary file 1 — Supplementary Information. [file 41598_2024_56794_MOESM1_ESM.zip › Supplementary/datasets/224.png]

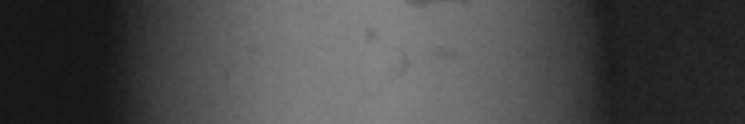

Supplement: Supplementary file 1 — Supplementary Information. [file 41598_2024_56794_MOESM1_ESM.zip › Supplementary/datasets/225.png]

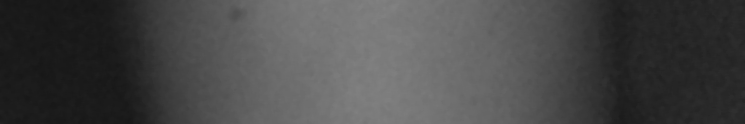

Supplement: Supplementary file 1 — Supplementary Information. [file 41598_2024_56794_MOESM1_ESM.zip › Supplementary/datasets/226.png]

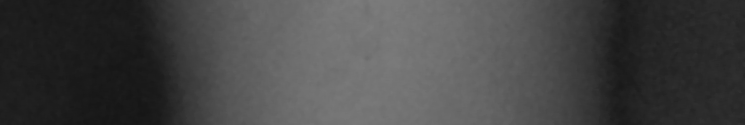

Supplement: Supplementary file 1 — Supplementary Information. [file 41598_2024_56794_MOESM1_ESM.zip › Supplementary/datasets/227.png]

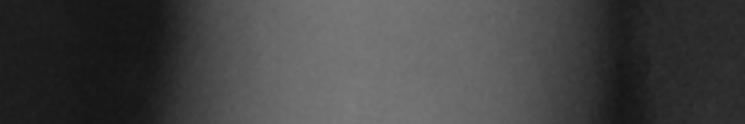

Supplement: Supplementary file 1 — Supplementary Information. [file 41598_2024_56794_MOESM1_ESM.zip › Supplementary/datasets/228.png]

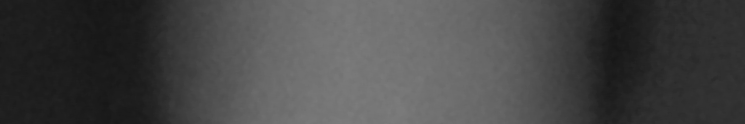

Supplement: Supplementary file 1 — Supplementary Information. [file 41598_2024_56794_MOESM1_ESM.zip › Supplementary/datasets/229.png]

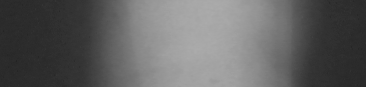

Supplement: Supplementary file 1 — Supplementary Information. [file 41598_2024_56794_MOESM1_ESM.zip › Supplementary/datasets/23.png]

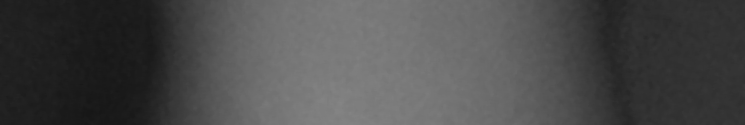

Supplement: Supplementary file 1 — Supplementary Information. [file 41598_2024_56794_MOESM1_ESM.zip › Supplementary/datasets/230.png]

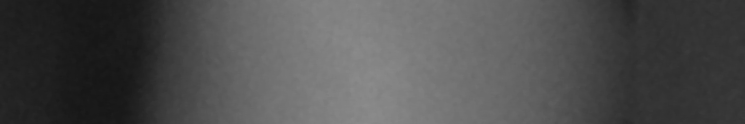

Supplement: Supplementary file 1 — Supplementary Information. [file 41598_2024_56794_MOESM1_ESM.zip › Supplementary/datasets/231.png]

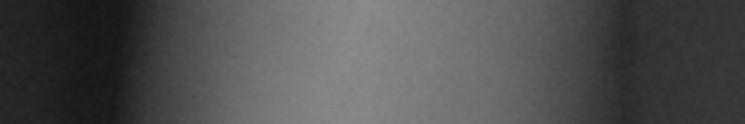

Supplement: Supplementary file 1 — Supplementary Information. [file 41598_2024_56794_MOESM1_ESM.zip › Supplementary/datasets/232.png]

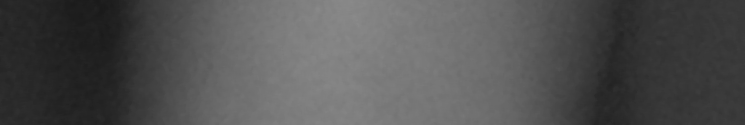

Supplement: Supplementary file 1 — Supplementary Information. [file 41598_2024_56794_MOESM1_ESM.zip › Supplementary/datasets/233.png]

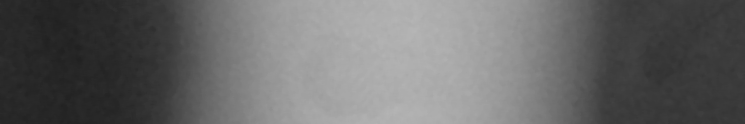

Supplement: Supplementary file 1 — Supplementary Information. [file 41598_2024_56794_MOESM1_ESM.zip › Supplementary/datasets/234.png]

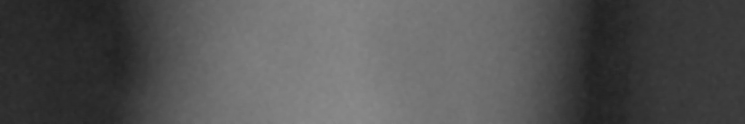

Supplement: Supplementary file 1 — Supplementary Information. [file 41598_2024_56794_MOESM1_ESM.zip › Supplementary/datasets/235.png]

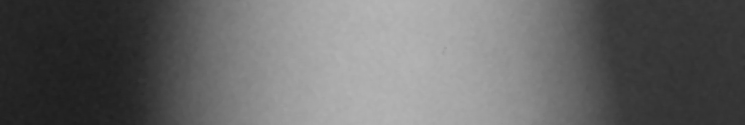

Supplement: Supplementary file 1 — Supplementary Information. [file 41598_2024_56794_MOESM1_ESM.zip › Supplementary/datasets/236.png]

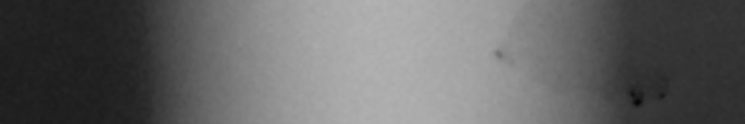

Supplement: Supplementary file 1 — Supplementary Information. [file 41598_2024_56794_MOESM1_ESM.zip › Supplementary/datasets/237.png]

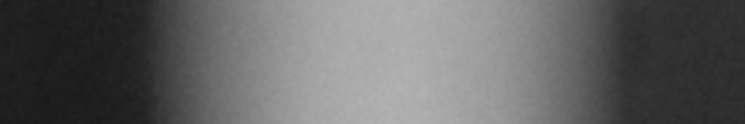

Supplement: Supplementary file 1 — Supplementary Information. [file 41598_2024_56794_MOESM1_ESM.zip › Supplementary/datasets/238.png]

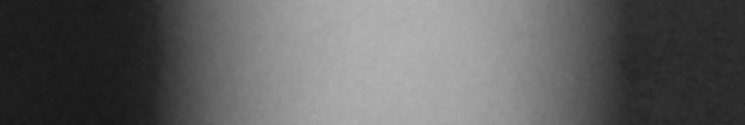

Supplement: Supplementary file 1 — Supplementary Information. [file 41598_2024_56794_MOESM1_ESM.zip › Supplementary/datasets/239.png]

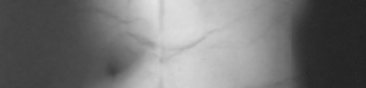

Supplement: Supplementary file 1 — Supplementary Information. [file 41598_2024_56794_MOESM1_ESM.zip › Supplementary/datasets/24.png]

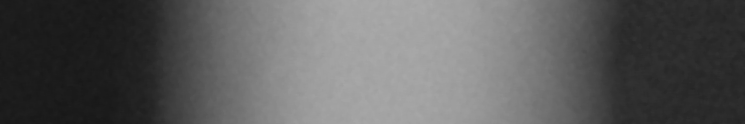

Supplement: Supplementary file 1 — Supplementary Information. [file 41598_2024_56794_MOESM1_ESM.zip › Supplementary/datasets/240.png]

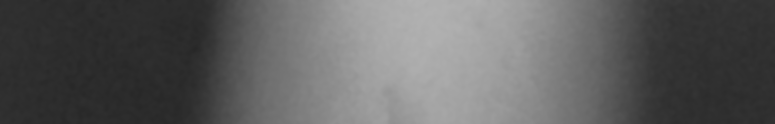

Supplement: Supplementary file 1 — Supplementary Information. [file 41598_2024_56794_MOESM1_ESM.zip › Supplementary/datasets/241.png]

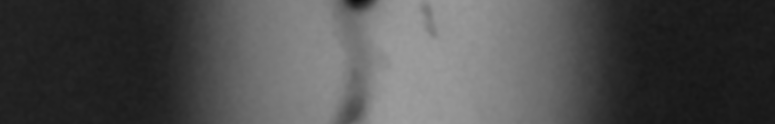

Supplement: Supplementary file 1 — Supplementary Information. [file 41598_2024_56794_MOESM1_ESM.zip › Supplementary/datasets/242.png]

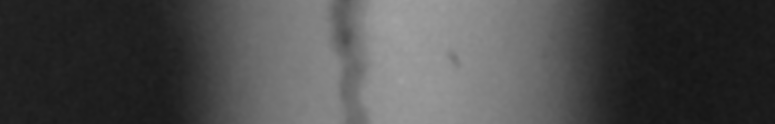

Supplement: Supplementary file 1 — Supplementary Information. [file 41598_2024_56794_MOESM1_ESM.zip › Supplementary/datasets/243.png]

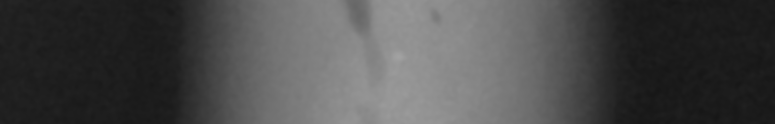

Supplement: Supplementary file 1 — Supplementary Information. [file 41598_2024_56794_MOESM1_ESM.zip › Supplementary/datasets/244.png]

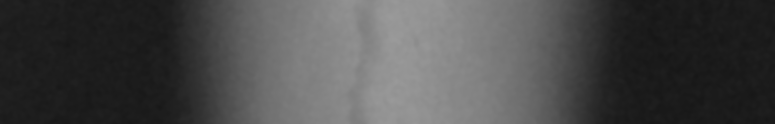

Supplement: Supplementary file 1 — Supplementary Information. [file 41598_2024_56794_MOESM1_ESM.zip › Supplementary/datasets/245.png]

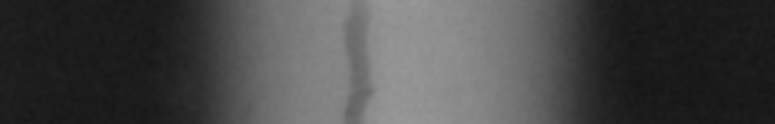

Supplement: Supplementary file 1 — Supplementary Information. [file 41598_2024_56794_MOESM1_ESM.zip › Supplementary/datasets/246.png]

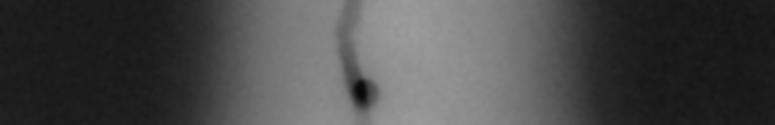

Supplement: Supplementary file 1 — Supplementary Information. [file 41598_2024_56794_MOESM1_ESM.zip › Supplementary/datasets/247.png]

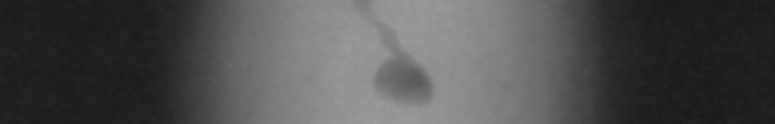

Supplement: Supplementary file 1 — Supplementary Information. [file 41598_2024_56794_MOESM1_ESM.zip › Supplementary/datasets/248.png]

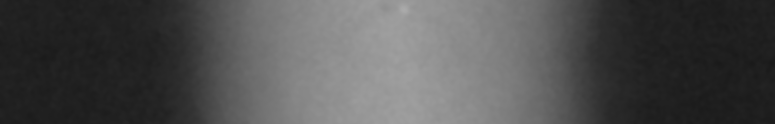

Supplement: Supplementary file 1 — Supplementary Information. [file 41598_2024_56794_MOESM1_ESM.zip › Supplementary/datasets/249.png]

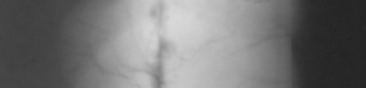

Supplement: Supplementary file 1 — Supplementary Information. [file 41598_2024_56794_MOESM1_ESM.zip › Supplementary/datasets/25.png]

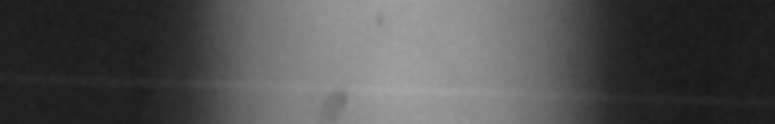

Supplement: Supplementary file 1 — Supplementary Information. [file 41598_2024_56794_MOESM1_ESM.zip › Supplementary/datasets/250.png]

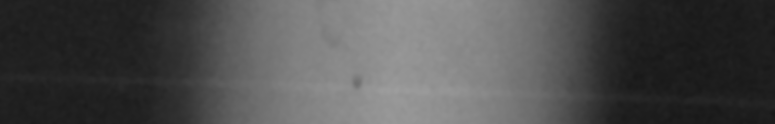

Supplement: Supplementary file 1 — Supplementary Information. [file 41598_2024_56794_MOESM1_ESM.zip › Supplementary/datasets/251.png]

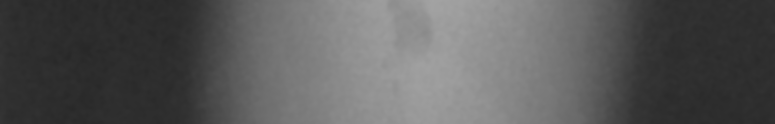

Supplement: Supplementary file 1 — Supplementary Information. [file 41598_2024_56794_MOESM1_ESM.zip › Supplementary/datasets/252.png]

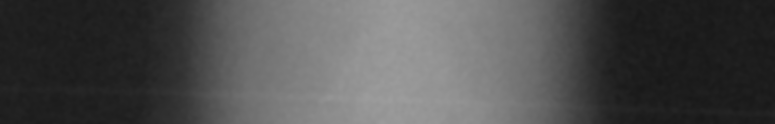

Supplement: Supplementary file 1 — Supplementary Information. [file 41598_2024_56794_MOESM1_ESM.zip › Supplementary/datasets/253.png]

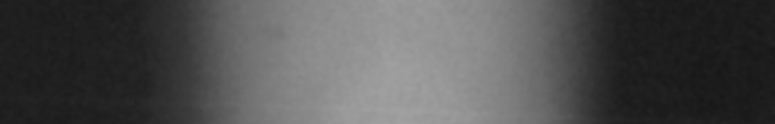

Supplement: Supplementary file 1 — Supplementary Information. [file 41598_2024_56794_MOESM1_ESM.zip › Supplementary/datasets/254.png]

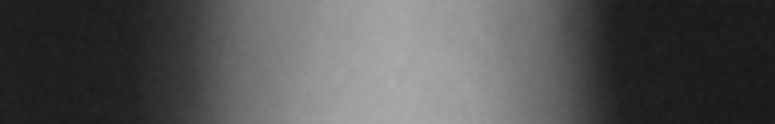

Supplement: Supplementary file 1 — Supplementary Information. [file 41598_2024_56794_MOESM1_ESM.zip › Supplementary/datasets/255.png]

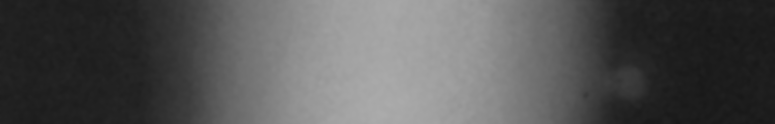

Supplement: Supplementary file 1 — Supplementary Information. [file 41598_2024_56794_MOESM1_ESM.zip › Supplementary/datasets/256.png]

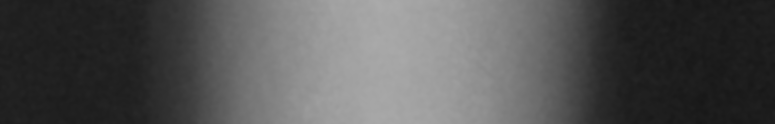

Supplement: Supplementary file 1 — Supplementary Information. [file 41598_2024_56794_MOESM1_ESM.zip › Supplementary/datasets/257.png]

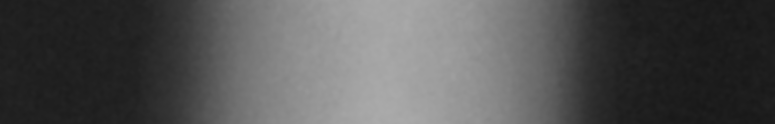

Supplement: Supplementary file 1 — Supplementary Information. [file 41598_2024_56794_MOESM1_ESM.zip › Supplementary/datasets/258.png]

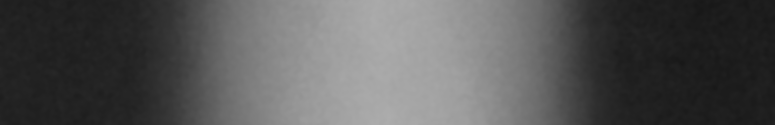

Supplement: Supplementary file 1 — Supplementary Information. [file 41598_2024_56794_MOESM1_ESM.zip › Supplementary/datasets/259.png]

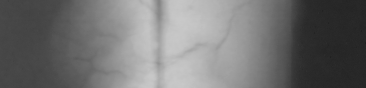

Supplement: Supplementary file 1 — Supplementary Information. [file 41598_2024_56794_MOESM1_ESM.zip › Supplementary/datasets/26.png]

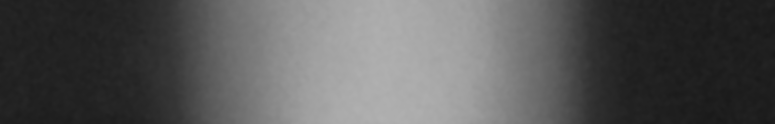

Supplement: Supplementary file 1 — Supplementary Information. [file 41598_2024_56794_MOESM1_ESM.zip › Supplementary/datasets/260.png]

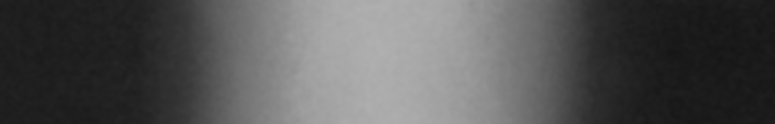

Supplement: Supplementary file 1 — Supplementary Information. [file 41598_2024_56794_MOESM1_ESM.zip › Supplementary/datasets/261.png]

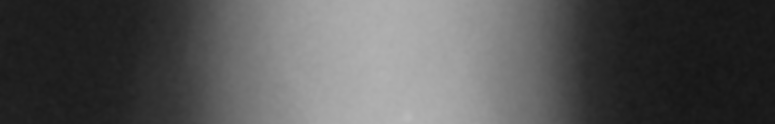

Supplement: Supplementary file 1 — Supplementary Information. [file 41598_2024_56794_MOESM1_ESM.zip › Supplementary/datasets/262.png]

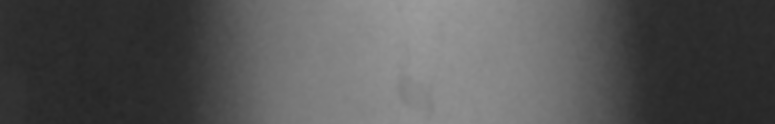

Supplement: Supplementary file 1 — Supplementary Information. [file 41598_2024_56794_MOESM1_ESM.zip › Supplementary/datasets/263.png]

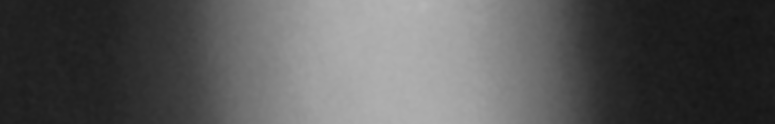

Supplement: Supplementary file 1 — Supplementary Information. [file 41598_2024_56794_MOESM1_ESM.zip › Supplementary/datasets/264.png]

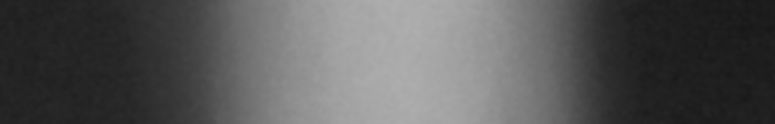

Supplement: Supplementary file 1 — Supplementary Information. [file 41598_2024_56794_MOESM1_ESM.zip › Supplementary/datasets/265.png]

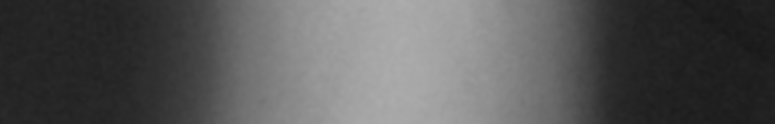

Supplement: Supplementary file 1 — Supplementary Information. [file 41598_2024_56794_MOESM1_ESM.zip › Supplementary/datasets/266.png]

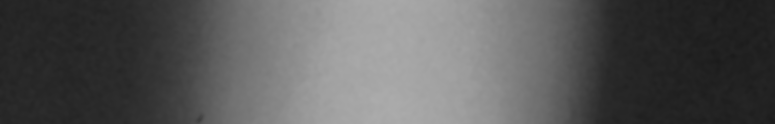

Supplement: Supplementary file 1 — Supplementary Information. [file 41598_2024_56794_MOESM1_ESM.zip › Supplementary/datasets/267.png]

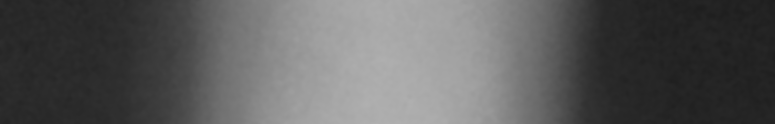

Supplement: Supplementary file 1 — Supplementary Information. [file 41598_2024_56794_MOESM1_ESM.zip › Supplementary/datasets/268.png]

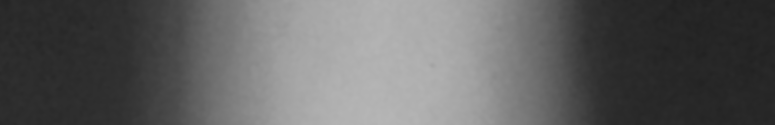

Supplement: Supplementary file 1 — Supplementary Information. [file 41598_2024_56794_MOESM1_ESM.zip › Supplementary/datasets/269.png]

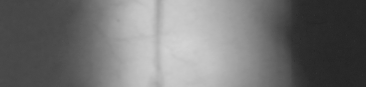

Supplement: Supplementary file 1 — Supplementary Information. [file 41598_2024_56794_MOESM1_ESM.zip › Supplementary/datasets/27.png]

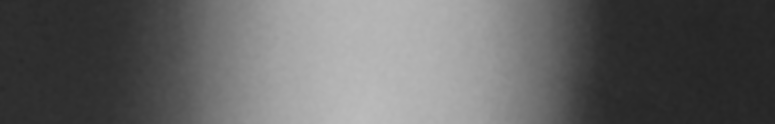

Supplement: Supplementary file 1 — Supplementary Information. [file 41598_2024_56794_MOESM1_ESM.zip › Supplementary/datasets/270.png]

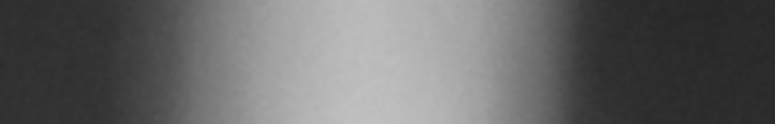

Supplement: Supplementary file 1 — Supplementary Information. [file 41598_2024_56794_MOESM1_ESM.zip › Supplementary/datasets/271.png]

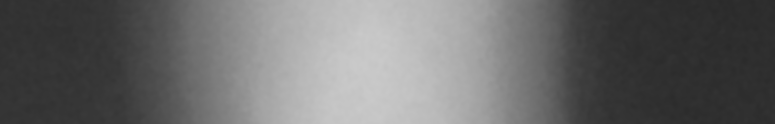

Supplement: Supplementary file 1 — Supplementary Information. [file 41598_2024_56794_MOESM1_ESM.zip › Supplementary/datasets/272.png]

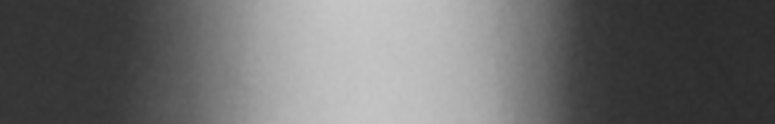

Supplement: Supplementary file 1 — Supplementary Information. [file 41598_2024_56794_MOESM1_ESM.zip › Supplementary/datasets/273.png]

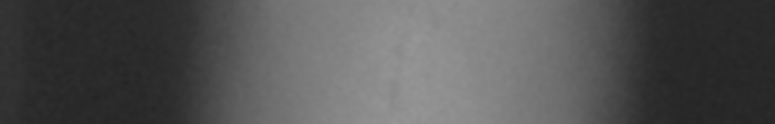

Supplement: Supplementary file 1 — Supplementary Information. [file 41598_2024_56794_MOESM1_ESM.zip › Supplementary/datasets/274.png]

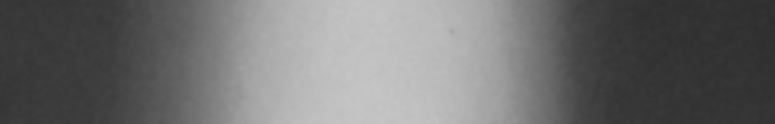

Supplement: Supplementary file 1 — Supplementary Information. [file 41598_2024_56794_MOESM1_ESM.zip › Supplementary/datasets/275.png]

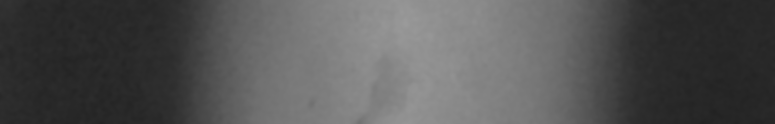

Supplement: Supplementary file 1 — Supplementary Information. [file 41598_2024_56794_MOESM1_ESM.zip › Supplementary/datasets/276.png]

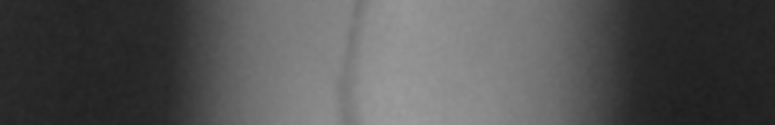

Supplement: Supplementary file 1 — Supplementary Information. [file 41598_2024_56794_MOESM1_ESM.zip › Supplementary/datasets/277.png]

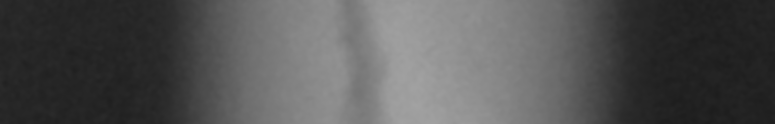

Supplement: Supplementary file 1 — Supplementary Information. [file 41598_2024_56794_MOESM1_ESM.zip › Supplementary/datasets/278.png]
