# Supplementary figures and images for: A new method for deep learning detection of defects in X-ray images of pressure vessel welds (part 1 of 2)
Source: Sci Rep. 2024 Mar 15;14:6312. doi: 10.1038/s41598-024-56794-9 (PMC10943115; doi:10.1038/s41598-024-56794-9)

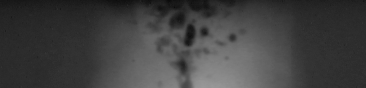

Supplement: Supplementary file 1 — Supplementary Information. [file 41598_2024_56794_MOESM1_ESM.zip › Supplementary/datasets/1.png]

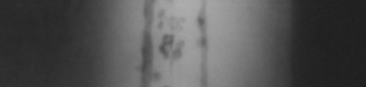

Supplement: Supplementary file 1 — Supplementary Information. [file 41598_2024_56794_MOESM1_ESM.zip › Supplementary/datasets/10.png]

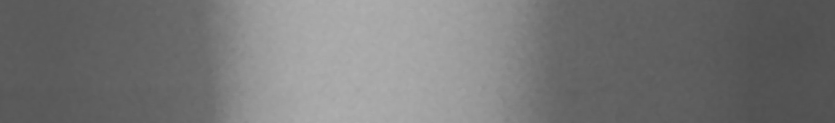

Supplement: Supplementary file 1 — Supplementary Information. [file 41598_2024_56794_MOESM1_ESM.zip › Supplementary/datasets/100.png]

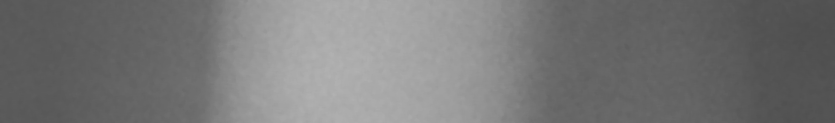

Supplement: Supplementary file 1 — Supplementary Information. [file 41598_2024_56794_MOESM1_ESM.zip › Supplementary/datasets/101.png]

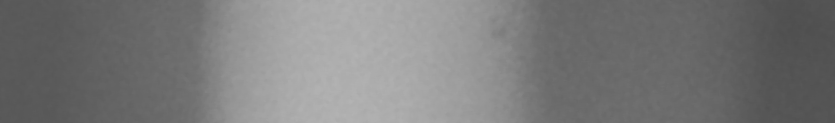

Supplement: Supplementary file 1 — Supplementary Information. [file 41598_2024_56794_MOESM1_ESM.zip › Supplementary/datasets/102.png]

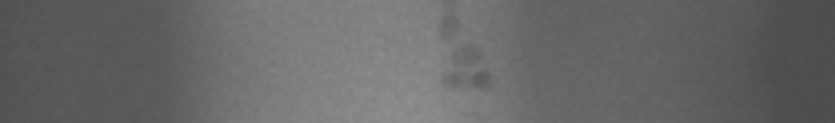

Supplement: Supplementary file 1 — Supplementary Information. [file 41598_2024_56794_MOESM1_ESM.zip › Supplementary/datasets/103.png]

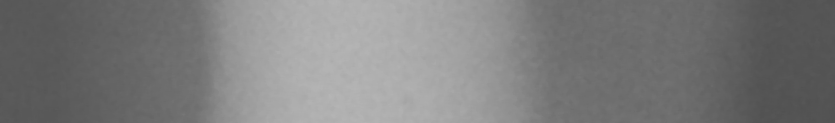

Supplement: Supplementary file 1 — Supplementary Information. [file 41598_2024_56794_MOESM1_ESM.zip › Supplementary/datasets/104.png]

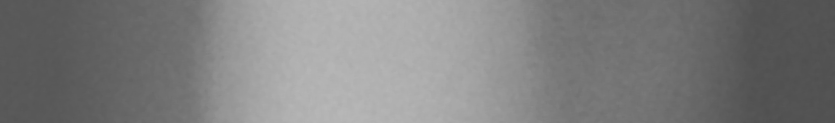

Supplement: Supplementary file 1 — Supplementary Information. [file 41598_2024_56794_MOESM1_ESM.zip › Supplementary/datasets/105.png]

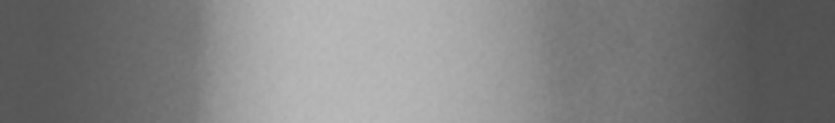

Supplement: Supplementary file 1 — Supplementary Information. [file 41598_2024_56794_MOESM1_ESM.zip › Supplementary/datasets/106.png]

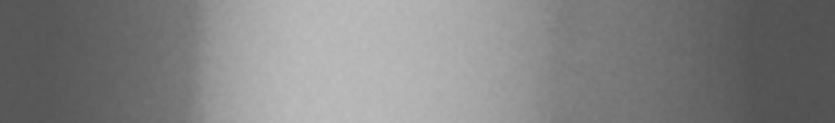

Supplement: Supplementary file 1 — Supplementary Information. [file 41598_2024_56794_MOESM1_ESM.zip › Supplementary/datasets/107.png]

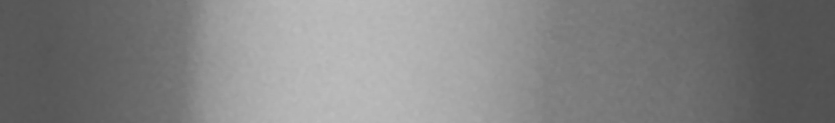

Supplement: Supplementary file 1 — Supplementary Information. [file 41598_2024_56794_MOESM1_ESM.zip › Supplementary/datasets/108.png]

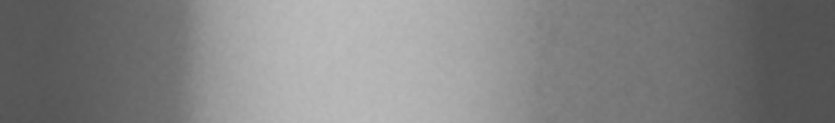

Supplement: Supplementary file 1 — Supplementary Information. [file 41598_2024_56794_MOESM1_ESM.zip › Supplementary/datasets/109.png]

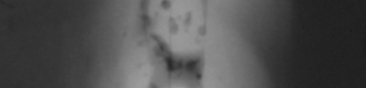

Supplement: Supplementary file 1 — Supplementary Information. [file 41598_2024_56794_MOESM1_ESM.zip › Supplementary/datasets/11.png]

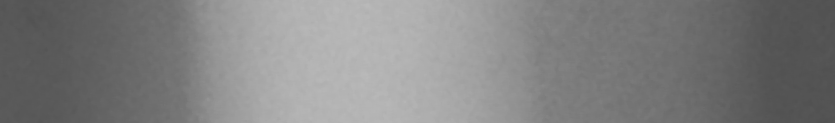

Supplement: Supplementary file 1 — Supplementary Information. [file 41598_2024_56794_MOESM1_ESM.zip › Supplementary/datasets/110.png]

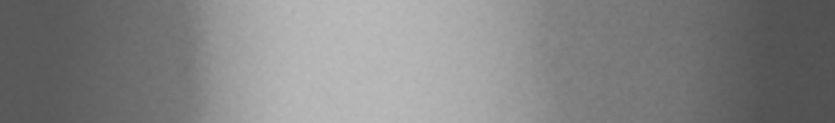

Supplement: Supplementary file 1 — Supplementary Information. [file 41598_2024_56794_MOESM1_ESM.zip › Supplementary/datasets/111.png]

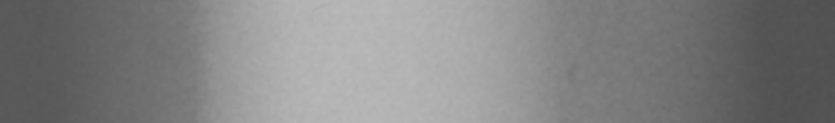

Supplement: Supplementary file 1 — Supplementary Information. [file 41598_2024_56794_MOESM1_ESM.zip › Supplementary/datasets/112.png]

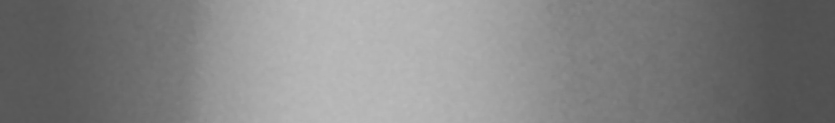

Supplement: Supplementary file 1 — Supplementary Information. [file 41598_2024_56794_MOESM1_ESM.zip › Supplementary/datasets/113.png]

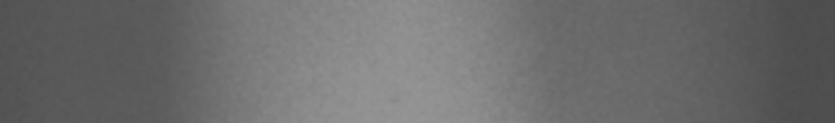

Supplement: Supplementary file 1 — Supplementary Information. [file 41598_2024_56794_MOESM1_ESM.zip › Supplementary/datasets/114.png]

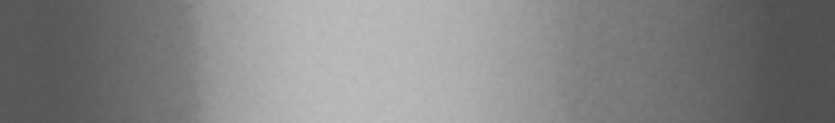

Supplement: Supplementary file 1 — Supplementary Information. [file 41598_2024_56794_MOESM1_ESM.zip › Supplementary/datasets/115.png]

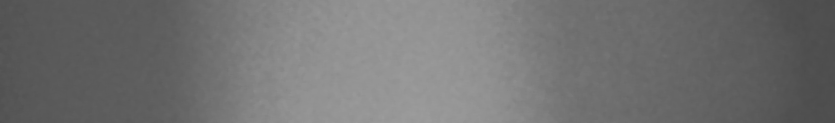

Supplement: Supplementary file 1 — Supplementary Information. [file 41598_2024_56794_MOESM1_ESM.zip › Supplementary/datasets/116.png]

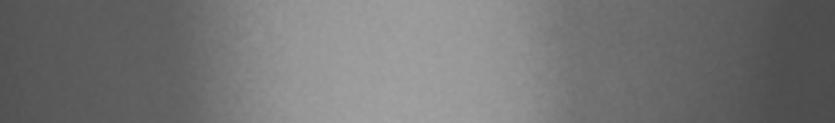

Supplement: Supplementary file 1 — Supplementary Information. [file 41598_2024_56794_MOESM1_ESM.zip › Supplementary/datasets/117.png]

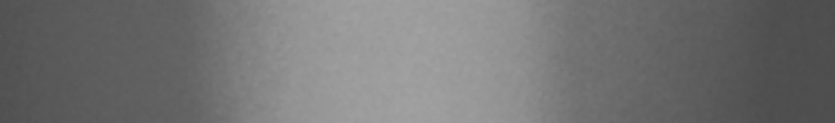

Supplement: Supplementary file 1 — Supplementary Information. [file 41598_2024_56794_MOESM1_ESM.zip › Supplementary/datasets/118.png]

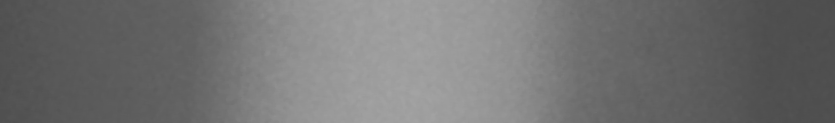

Supplement: Supplementary file 1 — Supplementary Information. [file 41598_2024_56794_MOESM1_ESM.zip › Supplementary/datasets/119.png]

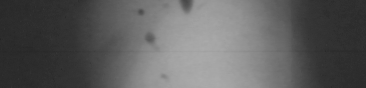

Supplement: Supplementary file 1 — Supplementary Information. [file 41598_2024_56794_MOESM1_ESM.zip › Supplementary/datasets/12.png]

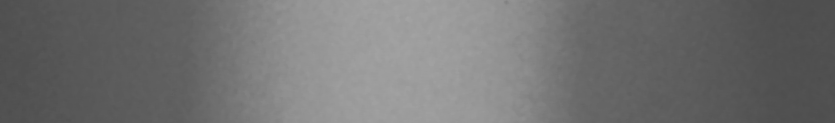

Supplement: Supplementary file 1 — Supplementary Information. [file 41598_2024_56794_MOESM1_ESM.zip › Supplementary/datasets/120.png]

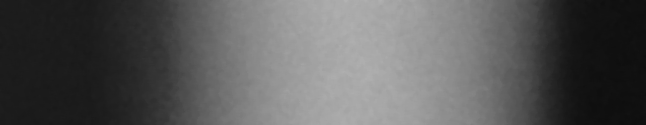

Supplement: Supplementary file 1 — Supplementary Information. [file 41598_2024_56794_MOESM1_ESM.zip › Supplementary/datasets/121.png]

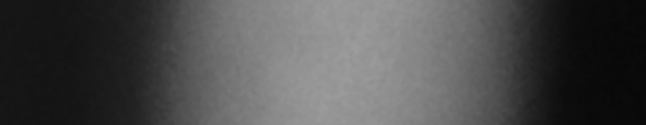

Supplement: Supplementary file 1 — Supplementary Information. [file 41598_2024_56794_MOESM1_ESM.zip › Supplementary/datasets/122.png]

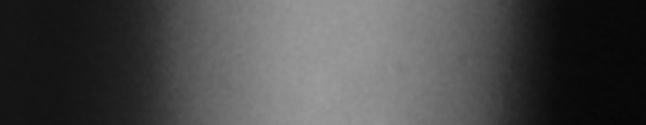

Supplement: Supplementary file 1 — Supplementary Information. [file 41598_2024_56794_MOESM1_ESM.zip › Supplementary/datasets/123.png]

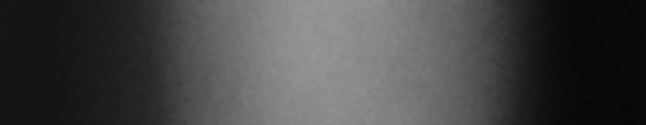

Supplement: Supplementary file 1 — Supplementary Information. [file 41598_2024_56794_MOESM1_ESM.zip › Supplementary/datasets/124.png]

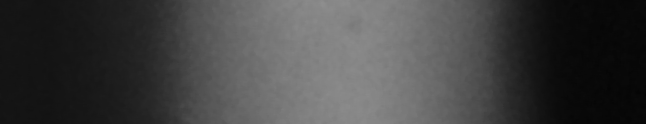

Supplement: Supplementary file 1 — Supplementary Information. [file 41598_2024_56794_MOESM1_ESM.zip › Supplementary/datasets/125.png]

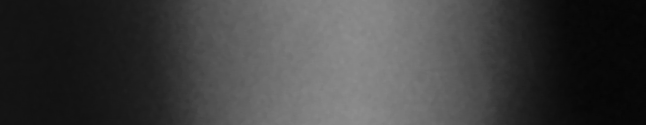

Supplement: Supplementary file 1 — Supplementary Information. [file 41598_2024_56794_MOESM1_ESM.zip › Supplementary/datasets/126.png]

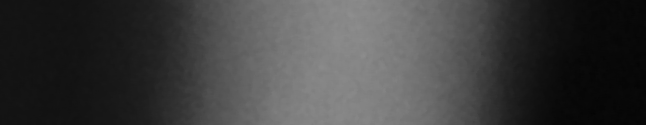

Supplement: Supplementary file 1 — Supplementary Information. [file 41598_2024_56794_MOESM1_ESM.zip › Supplementary/datasets/127.png]

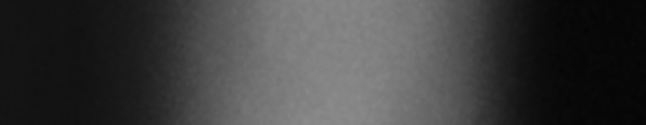

Supplement: Supplementary file 1 — Supplementary Information. [file 41598_2024_56794_MOESM1_ESM.zip › Supplementary/datasets/128.png]

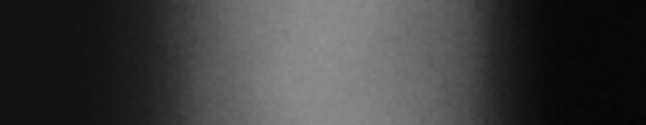

Supplement: Supplementary file 1 — Supplementary Information. [file 41598_2024_56794_MOESM1_ESM.zip › Supplementary/datasets/129.png]

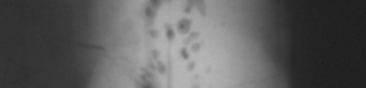

Supplement: Supplementary file 1 — Supplementary Information. [file 41598_2024_56794_MOESM1_ESM.zip › Supplementary/datasets/13.png]

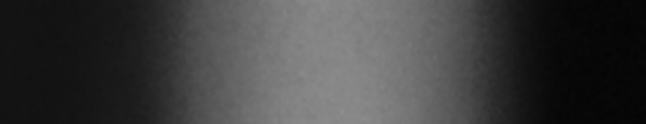

Supplement: Supplementary file 1 — Supplementary Information. [file 41598_2024_56794_MOESM1_ESM.zip › Supplementary/datasets/130.png]

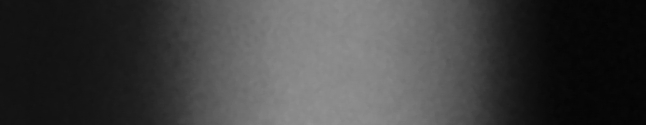

Supplement: Supplementary file 1 — Supplementary Information. [file 41598_2024_56794_MOESM1_ESM.zip › Supplementary/datasets/131.png]

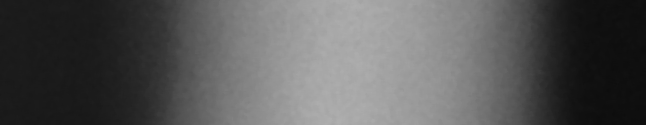

Supplement: Supplementary file 1 — Supplementary Information. [file 41598_2024_56794_MOESM1_ESM.zip › Supplementary/datasets/132.png]

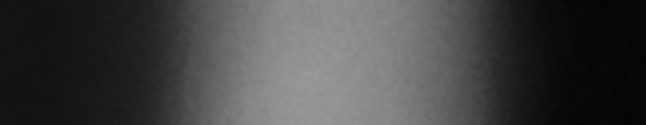

Supplement: Supplementary file 1 — Supplementary Information. [file 41598_2024_56794_MOESM1_ESM.zip › Supplementary/datasets/133.png]

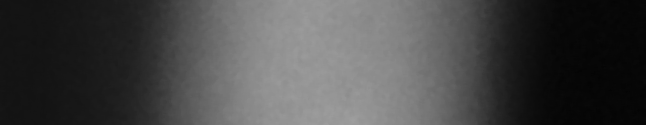

Supplement: Supplementary file 1 — Supplementary Information. [file 41598_2024_56794_MOESM1_ESM.zip › Supplementary/datasets/134.png]

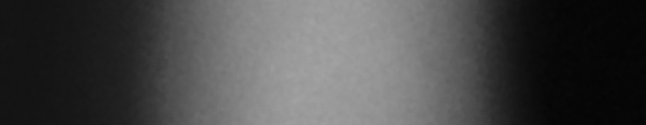

Supplement: Supplementary file 1 — Supplementary Information. [file 41598_2024_56794_MOESM1_ESM.zip › Supplementary/datasets/135.png]

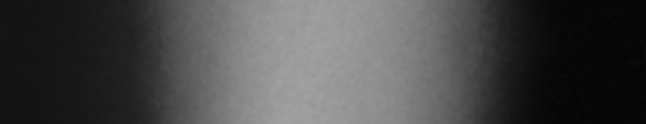

Supplement: Supplementary file 1 — Supplementary Information. [file 41598_2024_56794_MOESM1_ESM.zip › Supplementary/datasets/136.png]

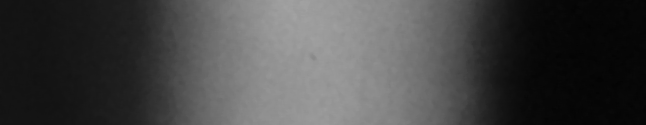

Supplement: Supplementary file 1 — Supplementary Information. [file 41598_2024_56794_MOESM1_ESM.zip › Supplementary/datasets/137.png]

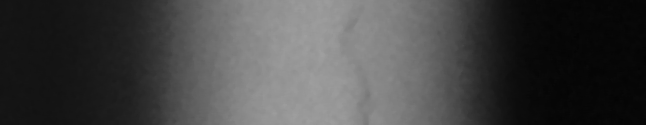

Supplement: Supplementary file 1 — Supplementary Information. [file 41598_2024_56794_MOESM1_ESM.zip › Supplementary/datasets/138.png]

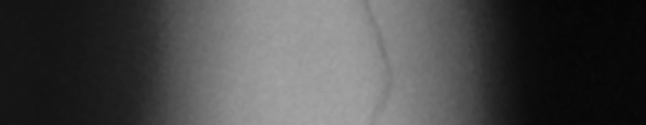

Supplement: Supplementary file 1 — Supplementary Information. [file 41598_2024_56794_MOESM1_ESM.zip › Supplementary/datasets/139.png]

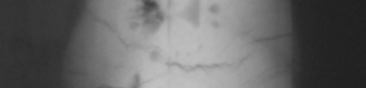

Supplement: Supplementary file 1 — Supplementary Information. [file 41598_2024_56794_MOESM1_ESM.zip › Supplementary/datasets/14.png]

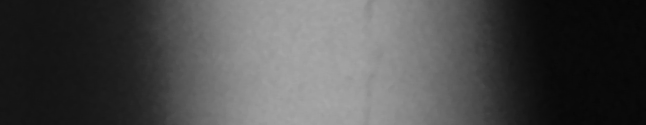

Supplement: Supplementary file 1 — Supplementary Information. [file 41598_2024_56794_MOESM1_ESM.zip › Supplementary/datasets/140.png]

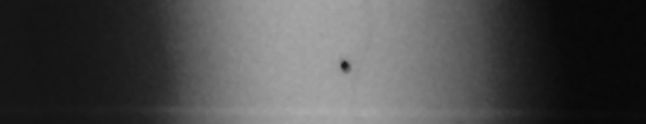

Supplement: Supplementary file 1 — Supplementary Information. [file 41598_2024_56794_MOESM1_ESM.zip › Supplementary/datasets/141.png]

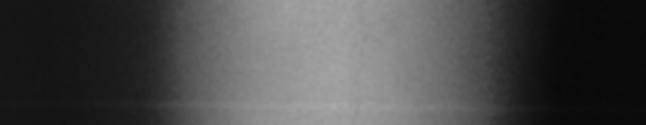

Supplement: Supplementary file 1 — Supplementary Information. [file 41598_2024_56794_MOESM1_ESM.zip › Supplementary/datasets/142.png]

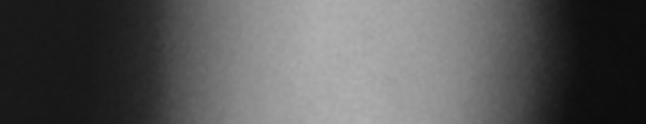

Supplement: Supplementary file 1 — Supplementary Information. [file 41598_2024_56794_MOESM1_ESM.zip › Supplementary/datasets/143.png]

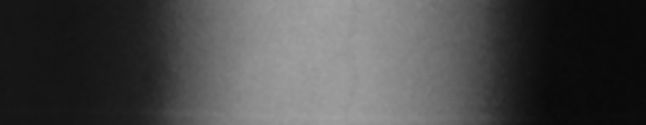

Supplement: Supplementary file 1 — Supplementary Information. [file 41598_2024_56794_MOESM1_ESM.zip › Supplementary/datasets/144.png]

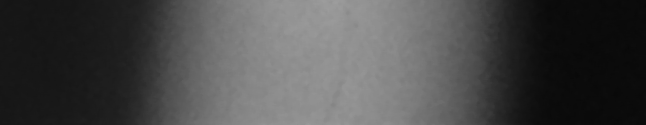

Supplement: Supplementary file 1 — Supplementary Information. [file 41598_2024_56794_MOESM1_ESM.zip › Supplementary/datasets/145.png]

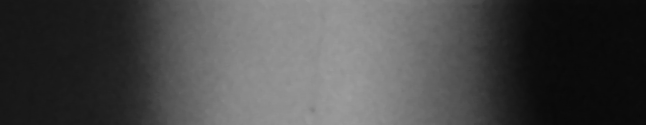

Supplement: Supplementary file 1 — Supplementary Information. [file 41598_2024_56794_MOESM1_ESM.zip › Supplementary/datasets/146.png]

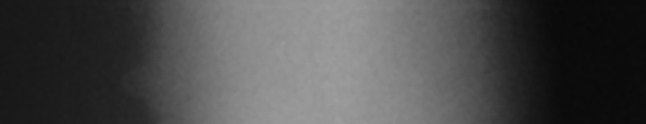

Supplement: Supplementary file 1 — Supplementary Information. [file 41598_2024_56794_MOESM1_ESM.zip › Supplementary/datasets/147.png]

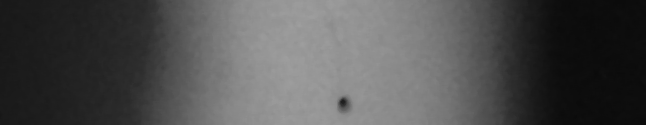

Supplement: Supplementary file 1 — Supplementary Information. [file 41598_2024_56794_MOESM1_ESM.zip › Supplementary/datasets/148.png]

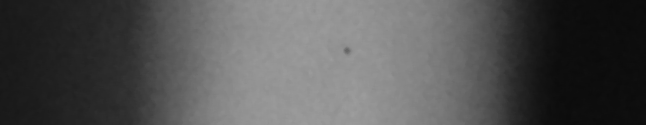

Supplement: Supplementary file 1 — Supplementary Information. [file 41598_2024_56794_MOESM1_ESM.zip › Supplementary/datasets/149.png]

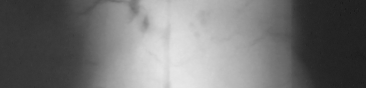

Supplement: Supplementary file 1 — Supplementary Information. [file 41598_2024_56794_MOESM1_ESM.zip › Supplementary/datasets/15.png]

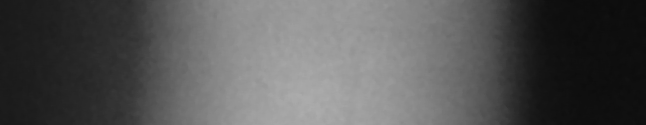

Supplement: Supplementary file 1 — Supplementary Information. [file 41598_2024_56794_MOESM1_ESM.zip › Supplementary/datasets/150.png]

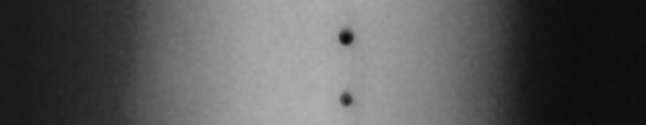

Supplement: Supplementary file 1 — Supplementary Information. [file 41598_2024_56794_MOESM1_ESM.zip › Supplementary/datasets/151.png]

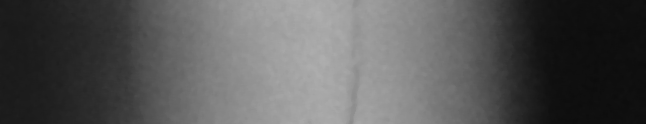

Supplement: Supplementary file 1 — Supplementary Information. [file 41598_2024_56794_MOESM1_ESM.zip › Supplementary/datasets/152.png]

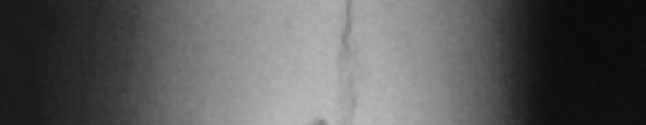

Supplement: Supplementary file 1 — Supplementary Information. [file 41598_2024_56794_MOESM1_ESM.zip › Supplementary/datasets/153.png]

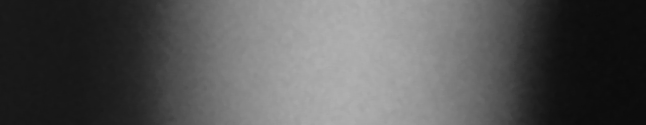

Supplement: Supplementary file 1 — Supplementary Information. [file 41598_2024_56794_MOESM1_ESM.zip › Supplementary/datasets/154.png]

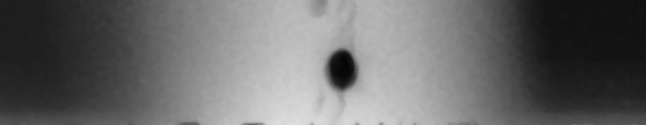

Supplement: Supplementary file 1 — Supplementary Information. [file 41598_2024_56794_MOESM1_ESM.zip › Supplementary/datasets/155.png]

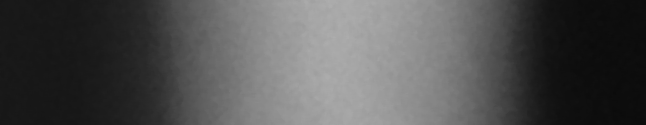

Supplement: Supplementary file 1 — Supplementary Information. [file 41598_2024_56794_MOESM1_ESM.zip › Supplementary/datasets/156.png]

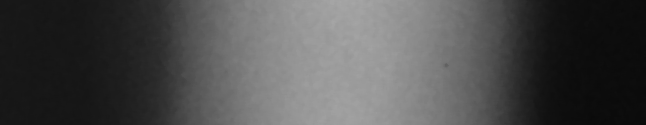

Supplement: Supplementary file 1 — Supplementary Information. [file 41598_2024_56794_MOESM1_ESM.zip › Supplementary/datasets/157.png]

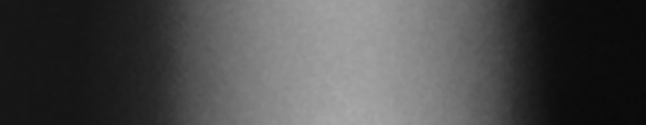

Supplement: Supplementary file 1 — Supplementary Information. [file 41598_2024_56794_MOESM1_ESM.zip › Supplementary/datasets/158.png]

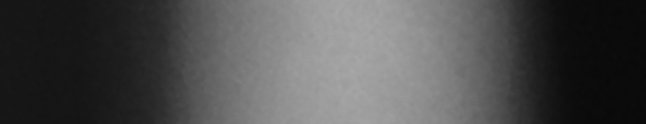

Supplement: Supplementary file 1 — Supplementary Information. [file 41598_2024_56794_MOESM1_ESM.zip › Supplementary/datasets/159.png]

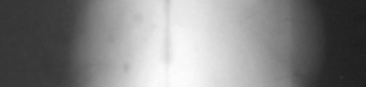

Supplement: Supplementary file 1 — Supplementary Information. [file 41598_2024_56794_MOESM1_ESM.zip › Supplementary/datasets/16.png]

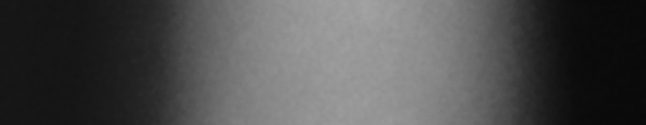

Supplement: Supplementary file 1 — Supplementary Information. [file 41598_2024_56794_MOESM1_ESM.zip › Supplementary/datasets/160.png]

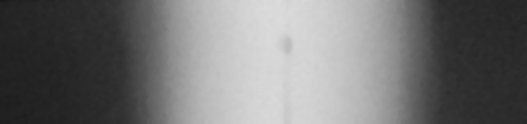

Supplement: Supplementary file 1 — Supplementary Information. [file 41598_2024_56794_MOESM1_ESM.zip › Supplementary/datasets/161.png]

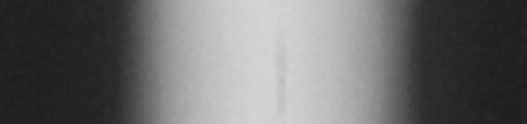

Supplement: Supplementary file 1 — Supplementary Information. [file 41598_2024_56794_MOESM1_ESM.zip › Supplementary/datasets/162.png]

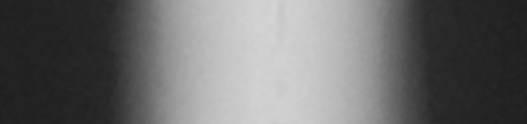

Supplement: Supplementary file 1 — Supplementary Information. [file 41598_2024_56794_MOESM1_ESM.zip › Supplementary/datasets/163.png]

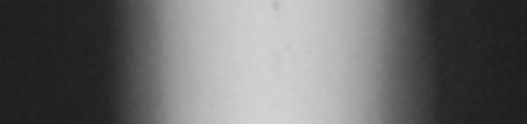

Supplement: Supplementary file 1 — Supplementary Information. [file 41598_2024_56794_MOESM1_ESM.zip › Supplementary/datasets/164.png]

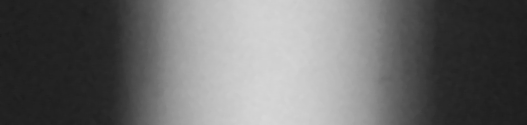

Supplement: Supplementary file 1 — Supplementary Information. [file 41598_2024_56794_MOESM1_ESM.zip › Supplementary/datasets/165.png]

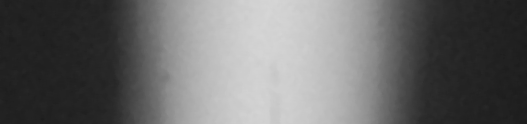

Supplement: Supplementary file 1 — Supplementary Information. [file 41598_2024_56794_MOESM1_ESM.zip › Supplementary/datasets/166.png]

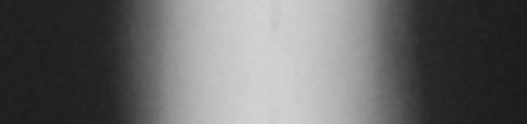

Supplement: Supplementary file 1 — Supplementary Information. [file 41598_2024_56794_MOESM1_ESM.zip › Supplementary/datasets/167.png]

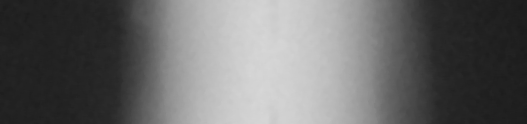

Supplement: Supplementary file 1 — Supplementary Information. [file 41598_2024_56794_MOESM1_ESM.zip › Supplementary/datasets/168.png]

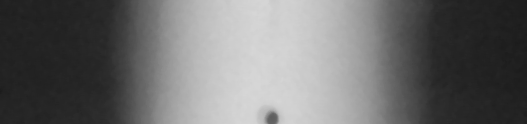

Supplement: Supplementary file 1 — Supplementary Information. [file 41598_2024_56794_MOESM1_ESM.zip › Supplementary/datasets/169.png]

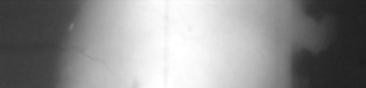

Supplement: Supplementary file 1 — Supplementary Information. [file 41598_2024_56794_MOESM1_ESM.zip › Supplementary/datasets/17.png]

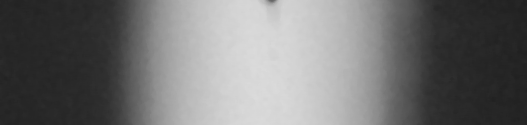

Supplement: Supplementary file 1 — Supplementary Information. [file 41598_2024_56794_MOESM1_ESM.zip › Supplementary/datasets/170.png]

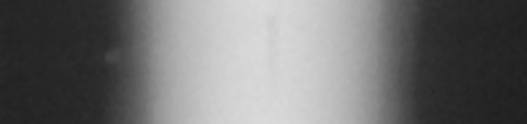

Supplement: Supplementary file 1 — Supplementary Information. [file 41598_2024_56794_MOESM1_ESM.zip › Supplementary/datasets/171.png]

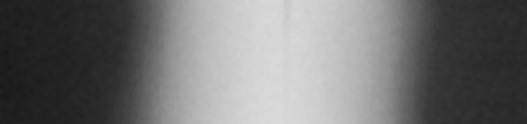

Supplement: Supplementary file 1 — Supplementary Information. [file 41598_2024_56794_MOESM1_ESM.zip › Supplementary/datasets/172.png]

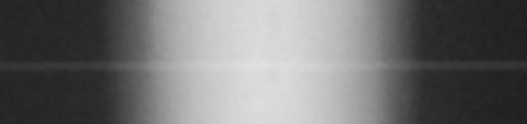

Supplement: Supplementary file 1 — Supplementary Information. [file 41598_2024_56794_MOESM1_ESM.zip › Supplementary/datasets/173.png]

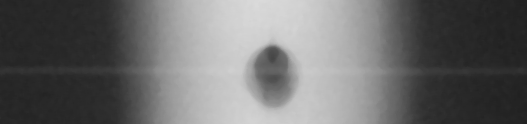

Supplement: Supplementary file 1 — Supplementary Information. [file 41598_2024_56794_MOESM1_ESM.zip › Supplementary/datasets/174.png]

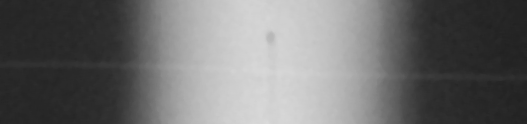

Supplement: Supplementary file 1 — Supplementary Information. [file 41598_2024_56794_MOESM1_ESM.zip › Supplementary/datasets/175.png]

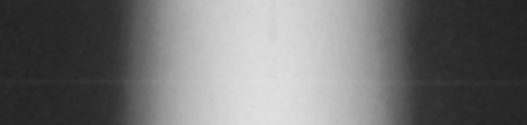

Supplement: Supplementary file 1 — Supplementary Information. [file 41598_2024_56794_MOESM1_ESM.zip › Supplementary/datasets/176.png]

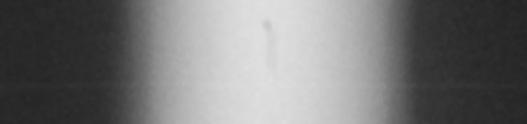

Supplement: Supplementary file 1 — Supplementary Information. [file 41598_2024_56794_MOESM1_ESM.zip › Supplementary/datasets/177.png]

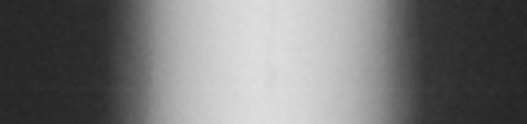

Supplement: Supplementary file 1 — Supplementary Information. [file 41598_2024_56794_MOESM1_ESM.zip › Supplementary/datasets/178.png]

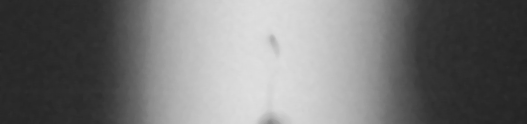

Supplement: Supplementary file 1 — Supplementary Information. [file 41598_2024_56794_MOESM1_ESM.zip › Supplementary/datasets/179.png]

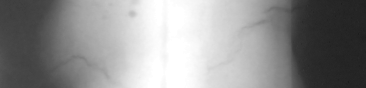

Supplement: Supplementary file 1 — Supplementary Information. [file 41598_2024_56794_MOESM1_ESM.zip › Supplementary/datasets/18.png]

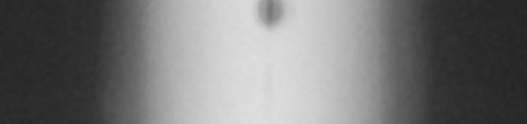

Supplement: Supplementary file 1 — Supplementary Information. [file 41598_2024_56794_MOESM1_ESM.zip › Supplementary/datasets/180.png]

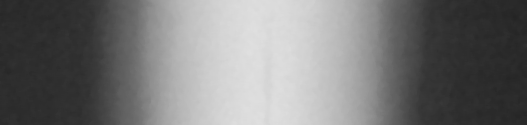

Supplement: Supplementary file 1 — Supplementary Information. [file 41598_2024_56794_MOESM1_ESM.zip › Supplementary/datasets/181.png]

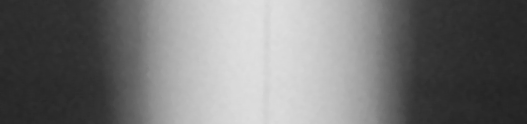

Supplement: Supplementary file 1 — Supplementary Information. [file 41598_2024_56794_MOESM1_ESM.zip › Supplementary/datasets/182.png]

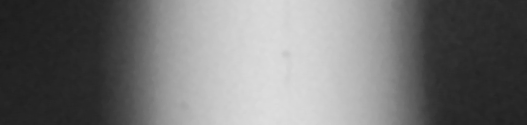

Supplement: Supplementary file 1 — Supplementary Information. [file 41598_2024_56794_MOESM1_ESM.zip › Supplementary/datasets/183.png]

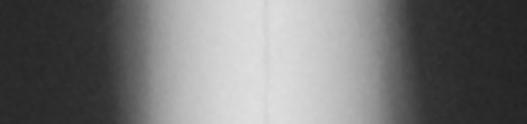

Supplement: Supplementary file 1 — Supplementary Information. [file 41598_2024_56794_MOESM1_ESM.zip › Supplementary/datasets/184.png]

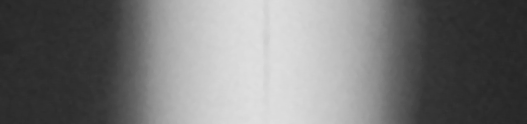

Supplement: Supplementary file 1 — Supplementary Information. [file 41598_2024_56794_MOESM1_ESM.zip › Supplementary/datasets/185.png]

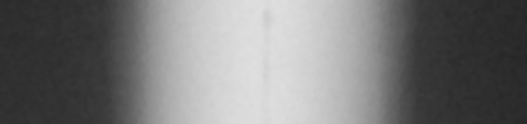

Supplement: Supplementary file 1 — Supplementary Information. [file 41598_2024_56794_MOESM1_ESM.zip › Supplementary/datasets/186.png]

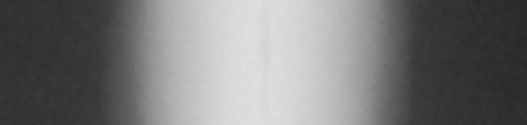

Supplement: Supplementary file 1 — Supplementary Information. [file 41598_2024_56794_MOESM1_ESM.zip › Supplementary/datasets/187.png]

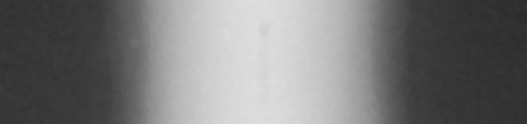

Supplement: Supplementary file 1 — Supplementary Information. [file 41598_2024_56794_MOESM1_ESM.zip › Supplementary/datasets/188.png]

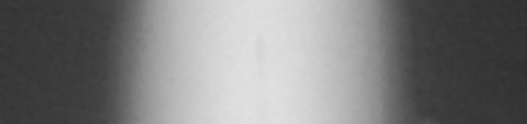

Supplement: Supplementary file 1 — Supplementary Information. [file 41598_2024_56794_MOESM1_ESM.zip › Supplementary/datasets/189.png]
